# Mitigating gut dysbiosis induced by biofilm-forming pathogens: therapeutic potential of LAB-derived bacteriocins

**DOI:** 10.3389/fmicb.2025.1721987

**Published:** 2026-01-12

**Authors:** Abinash Ravi, Ieshita Pan

**Affiliations:** Department of Medical Biotechnology, Institute of Biotechnology, Saveetha School of Engineering, Saveetha Institute of Medical and Technical Sciences, Chennai, Tamil Nadu, India

**Keywords:** antimicrobial activity, bacteriocins, gastrointestinal health, gut dysbiosis, gut microbiotamodulation, gut-brain axis

## Abstract

**Introduction:**

The gut-brain axis plays a critical role in bidirectional communication system connecting intestinal and neurological health. Imbalance in this system, often caused by gut dysbiosis and pathogenic biofilm formation, can result in inflammation, intestinal barrier dysfunction, and microbial imbalance.

**Methods:**

LAB isolates were screened for antimicrobial and antibiofilm activity against *Escherichia coli* and *Serratia marcescens*. The most effective strain, C82, was identified through morphological, biochemical and 16S rDNA sequencing. Bacteriocin production, and stability were assessed and optimized. The functional efficacy of the bacteriocin was tested in a zebrafish larval gut dysbiosis model.

**Results:**

*Lactiplantibacillus pentosus* C82, confirmed through 16S rDNA sequencing (NCBI GenBank submission ID: SUB14502111, accession number PP860573), demonstrated strong antimicrobial activity, with inhibition zones of 1.2 cm against *E. coli* and 1.4 cm against *S. marcescens*. The bacteriocin reduced inflammation and improved gut barrier integrity. It upregulated IL-10, Claudin-5a, ZO-1, Nfe2l2a, and Hmox1a, while downregulating TNF-*α*, csgD, and bsmA.

**Discussion and conclusion:**

These results establish *L. pentosus* C82 bacteriocin as a safe, stable, and potent natural antimicrobial agent with significant antibiofilm, antioxidative, and gut-protective effects. It shows promise as a bio-therapeutic candidate for restoring microbial balance, addressing gut dysbiosis, and influencing the gut-brain axis. However, the study was limited to short-term evaluation in zebrafish larvae, which may not fully represent the complexity of the mammalian gut. Further research involving long-term exposure and higher animal models is necessary to validate its therapeutic potential.

## Graphical abstract

**Figure:**
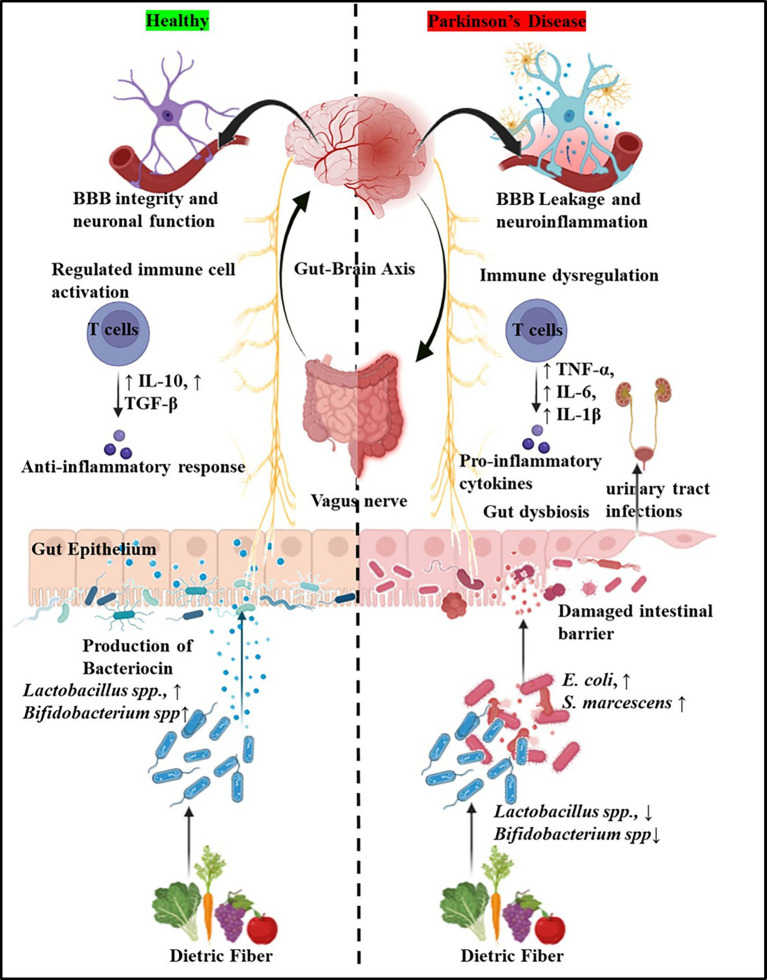

## Highlights

- Gut dysbiosis alters microbiota composition, leading to increased intestinal permeability, inflammation, and impaired gut-brain communication.
- Pathogenic biofilms enhance bacterial persistence, antimicrobial resistance, and gut inflammation, disrupting gut microbiota homeostasis.
- LAB-derived bacteriocins inhibit pathogens like *E. coli* and *Serratia marcescens*, reducing dysbiosis-related infections.
- Pathogenic biofilms, composed of EPS, proteins, and extracellular DNA, contribute to bacterial persistence, antimicrobial resistance, and gut microbiota imbalance.

## Introduction

The gut-brain axis (GBA) represents a dynamic and intricate communication network that connects the gastrointestinal tract with the central nervous system, playing a pivotal role in regulating neurological, metabolic, and immune functions (Carabotti et al., 2015; Lu et al., 2024). Disruption of this axis, commonly due to gut dysbiosis, can lead to inflammatory responses, increased intestinal permeability, and altered neuronal signaling, contributing to the pathogenesis of various neurological and gastrointestinal disorders (Ashique et al., 2024; Shen et al., 2025). One of the key factors driving gut dysbiosis is the formation of biofilms by pathogenic microorganisms, which not only shield bacteria from host defences and antimicrobial agents but also promote persistent infections and inflammation (Vestby et al., 2020; Montanari et al., 2025). These biofilms are primarily composed of an extracellular matrix containing exopolysaccharides (EPS), proteins, and extracellular DNA, enabling pathogens such as *Escherichia coli* and *Serratia marcescens* to adhere, colonize, and survive within the host gut environment (Saharan et al., 2024; Sharma and Rajpurohit, 2024).

*E. coli* and *S. marcescens* are opportunistic pathogens that can establish themselves in the host gut through specialized adaptations, promoting persistence and competition with the commensal microbiota. These bacteria possess potent adherence structures, including fimbriae, pili, and flagella, that enable them to attach firmly to intestinal epithelial cells and mucus. Their ability to form biofilms also confers protection against immune defences and antimicrobial agents (Orruño et al., 2014; Hoarau et al., 2016). Their versatile nature allows them to survive the unfavorable conditions caused by antibiotics, inflammation, or dietary shifts by exploiting diverse nutrient sources (Weiss and Hennet, 2017). By downregulating host immune responses or inducing localized inflammation, these pathogens gain a competitive advantage over beneficial species (Sina Rahme et al., 2022). Consequently, their expansion contributes to gut dysbiosis through mechanisms such as competitive overgrowth, epithelial barrier disruption, and promotion of inflammatory responses, often in synergy with other opportunistic microbes. Collectively, these strategies enable *E. coli* and *S. marcescens* to destabilize gut microbial communities, aggravating dysbiosis and predisposing the host to disease (Ochieng et al., 2014; Khan et al., 2021).

Gut dysbiosis remains a significant therapeutic challenge. Conventional interventions, such as antibiotics, dietary modifications, prebiotics, symbiotics, and fecal microbiota transplantation (FMT), are limited by reduced microbial diversity, resistance emergence, variable efficacy, and safety or regulatory concerns (Hou et al., 2025). Antibiotics eliminate both beneficial and harmful microbes, while FMT, though effective in specific contexts, carries risks of pathogen transmission (Kasar et al., 2025). Similarly, dietary and symbiotic approaches often yield inconsistent or short-lived benefits. In contrast, metabolites like organic acids and bacteriocins produced by lactic acid bacteria (LAB), especially *Lactobacillus* and *Bifidobacterium*, represent a safe and effective alternative with multifaceted mechanisms of action (Alagiakrishnan et al., 2024). There is growing interest in using LAB as a natural strategy to combat gut dysbiosis, due to their ability to produce antimicrobial peptides called bacteriocins (Anjana and Tiwari, 2022; Ismael et al., 2024). They restore microbial balance by competing with pathogens, enhancing epithelial barrier integrity, reducing inflammation, and modulating immune responses. Moreover, their bacteriocin production supports epithelial health and inhibits pathogen overgrowth. LAB-derived bacteriocins exhibit strong inhibitory activity against a wide range of pathogens, including *E. coli* and *S. marcescens*, making them promising candidates for restoring gut microbiota balance and preventing biofilm formation. In addition to their antimicrobial properties, certain LAB strains can produce EPS and short-chain fatty acids with health-promoting effects, such as strengthening the gut barrier and modulating the host immune response. These dual functionalities highlight the potential of LAB metabolites in developing novel biotherapeutic agents for maintaining gastrointestinal health (Ayed et al., 2024; Liang et al., 2024). Furthermore, due to their proven safety in food and clinical use, as well as broad effects on microbial, immune, and metabolic functions, LAB-derived metabolites emerge as a promising strategy for managing gut dysbiosis compared to more aggressive or uncertain alternatives (Hrncir, 2022; Kasar et al., 2025).

Fermented foods, especially fermented vegetables like cabbage (*Brassica oleracea*), are a great source of LAB. Cabbage naturally ferments well due to its nutrient composition, microbial community, and structural properties (Drašković Berger et al., 2020). Raw cabbage naturally harbors diverse LAB species, such as *Lactobacillus, Leuconostoc, Weissella,* and *Pediococcus*, that spontaneously drive fermentation without the need for external inoculation (Afriani et al., 2025). Cabbage contains sugars, vitamins, and minerals that support rapid LAB growth. These nutrients promote quick acidification and suppress harmful microbes, ensuring safe fermentation. Shredding and salting the cabbage enhances this process by creating an anaerobic and nutrient-rich environment. This creates a predictable shift in the dominant microbial population, from *Leuconostoc* and *Weissella* to *Lactobacillus*, leading to the development of a diverse LAB community (Touret et al., 2018). Fermented cabbage also produces beneficial metabolites, including organic acids and antioxidant compounds, which are reported in the literature to contribute to its probiotic benefits. Due to these qualities, cabbage was chosen in this study as a unique and naturally rich source for isolating LAB with potential therapeutic uses.

In this context, the present study focuses on isolating and characterizing bacteriocin-producing LAB from fermented cabbage and their effectiveness against biofilm-forming pathogens associated with gut dysbiosis. Specifically, the strain *Lactiplantibacillus pentosus* C82 was identified for its strong antimicrobial activity and bacteriocin production. Additionally, the efficacy of its bacteriocin was assessed using a zebrafish model of gut dysbiosis, providing insights into its potential therapeutic use in regulating inflammation, biofilm formation, and microbial imbalance.

## Materials and methods

### Sauerkraut fermentation

Fresh cabbage (*Brassica oleracea* var. *capitata*) was purchased from a local market in Chennai (13.0843° N, 80.2705° E). The cabbage was carefully cleaned by removing the outer leaves to prevent contamination, rinsed with cold water, and shredded into fine pieces to increase surface area. Approximately 350 g of shredded cabbage was placed in a sterile 3% sodium chloride solution and left to ferment in a sterile container at 37 °C for 35 days. The fermentation vessel was sealed to reduce oxygen exposure. Conditions such as high salt concentration, 70% moisture, and limited aeration were maintained to promote the selective growth of LAB (Touret et al., 2018).

### Isolation and identification of LAB

After 35 days of fermentation, 1 mL of the fermented brine was serially diluted up to 10^−3^. From this dilution, 100 μL was aseptically spread onto de Man, Rogosa, and Sharpe (MRS) agar plates using an L-shaped spreader. The plates were then incubated at 37 °C for 48 h to allow the LAB to grow. Pure LAB colonies were obtained through repeated sub-culturing and confirmed by colony morphology and Gram staining (Orji et al., 2019). MRS medium was initially chosen as the primary isolation medium because it is the gold standard for cultivating *Lactobacillus* species and provides optimal recovery of LAB from fermented plant matrices. Despite containing animal-derived components, MRS serves as a reliable baseline for isolating fastidious LAB before conducting medium optimization (Zahara et al., 2024).

### Agar well diffusion assay

The antimicrobial activity of the LAB isolates was assessed against *Escherichia coli* and *Serratia marcescens* using the agar well diffusion assay. Clinically confirmed pathogenic strains were obtained from the Department of Microbiology, at Saveetha Medical College and Hospital, Saveetha Nagar, Thandalam, Chennai – 602105, Tamil Nadu, India. Each pathogenic strain (100 μL) was uniformly spread onto separate nutrient agar plates. Once solidified, wells were aseptically punched using a sterile 200 μL micropipette tip. Subsequently, 20 μL of the cell-free supernatant from the LAB isolates was added to each well. Ampicillin (1 mg/mL) was used as the positive control. The plates were then incubated at 37 °C for 48 h, and the diameter of the inhibition zones was measured in millimeters (mm). LAB isolates that exhibited distinct inhibition zones were chosen for further identification (Dimkić et al., 2013; Nami et al., 2018).

### Screening and identification of selected isolate (C82)

#### Morphological characterization

The isolates were underwent microscopic and colony morphology analysis. Cell size and shape were determined using micrometry. Gram staining was performed to confirm the isolate’s Gram-positive nature (Agarwal and Sheikh, 2025).

#### Biochemical characterization

##### Catalase test

To test for catalase activity, a loopful of the bacterial culture was placed on a clean glass slide, and a few drops of 3% hydrogen peroxide were added. The formation of bubbles indicated catalase-positive samples, which were selected for further characterization tests (Ismail et al., 2018).

##### Gas production test

To differentiate between heterofermentative and homofermentative isolates, MRS medium was modified with three different pH levels: 2, 4, and 7. The isolates were inoculated into the medium and incubated at 37 °C for 48 h at 140 rpm. Gas production was monitored using Durham tubes, following the method described by Goa et al. (2022). Heterofermentative isolates were chosen for further testing.

##### Acid tolerance test

To assess acid tolerance, 1 mL of an overnight culture of each selected isolate (10^8^ CFU/mL) was inoculated into 10 mL of MRS broth adjusted to pH levels of 2, 4, and 7. The cultures were then incubated at 37 °C for 24 h with shaking at 140 rpm. Bacterial growth under each pH condition was evaluated by measuring the optical density (OD) at 600 nm using a spectrophotometer (Zaghloul and Ibrahim, 2022). Isolates were selected for further testing based on their survivability.

##### Bile salt tolerance

To determine bile resistance, the isolate was cultured in MRS broth supplemented with 0.3, 0.5, and 0.7% bile salts. After 24 h of incubation at 37 °C and 140 rpm, growth was measured spectrophotometrically at 600 nm and compared to the control (Zaghloul and Ibrahim, 2022). Isolates were chosen for further characterization tests based on their survivability.

##### Carbohydrate utilization

Carbohydrate utilization testing was conducted on the selected bacterial isolate to assess its ability to metabolize various carbohydrates, important for energy regulation. MRS broth containing phenol red indicator was supplemented individually with 1% of carbohydrates, including glucose, sucrose, lactose, maltose, fructose, and galactose. The broth was then inoculated with the selected isolate and incubated at 37 °C for 24–48 h. The presence of lactic acid in the fermented broth was confirmed using the ferric chloride assay (Borshchevskaya et al., 2016; Das et al., 2019).

##### Neutralization of cell-free supernatant

In order to determine whether the antimicrobial activity was a result of lactic acid or acidic pH, the cell-free supernatant (CFS) of *L. pentosus* C82 was adjusted to pH 6.5 ± 0.1 using sterile 1 M NaOH. The neutralized CFS was then filtered (0.22 μm) to remove any residual cells and used for agar well diffusion assays (Yaakoubi et al., 2009; Hacıoğlu and Türkyılmaz, 2024).

##### Catalase treatment of CFS

To investigate if hydrogen peroxide played a role in the antimicrobial effect, catalase (100 μg/mL) was added to freshly prepared CFS and incubated for 30 min at 37 °C. The catalase-treated CFS was then filter-sterilized before testing (El Ahmadi et al., 2025).

#### Molecular characterization

Genomic DNA was extracted using the HiPurA Bacterial DNA purification spin column kit (MB505-250PR, HiMedia, India) and checked on a 1% agarose gel electrophoresis. PCR amplification of the bacterial-specific 16S rRNA gene (1,500 bp) was carried out using primers F27 (5 ‘AGAGTTTGATCMTGGCTCAG 3’) and 1492R (5’ GGTTACCTTGTTACGACTT 3′) (Clarridge, 2004). The PCR reaction was performed in a 25 μL volume containing 12.5 μL EmeraldAmp GT PCR Master Mix, 2x (Takara Bio USA), 1 μL DNA template (50–100 ng), 1.25 μL (10 μM) of each primer (forward and reverse), and 9 μL of free-nuclease water. PCR amplification was performed using an Applied Biosystems Veriti Thermal Cycler as follows: denaturation at 94 °C for 5 min, followed by 34 cycles of 94 °C for 30 s, 55 °C for 30 s, and 72 °C for 1.30 min, and a final cycle at 72 °C for 7 min. The PCR products were detected by staining with GelRed Nucleic Acid Gel Stain on 1% agarose electrophoresis gel in (1X) TBE buffer and visualized under a UV transilluminator (Protein Simple Red Imager SA-1000). PCR product was purified using the Exonuclease I and Shrimp Alkaline Phosphatase Purification Kit (New England Biolabs, Inc) and cycle sequenced using the Big-Dye Terminator v.3.1 Cycle Sequencing Kit (Applied Biosystems, USA) with the following conditions: denaturation at 96 °C for 1 min followed by 28 cycles of 96 °C for 1 min, 50 °C for 5 s, and 60 °C for 4 min. The cycle sequenced amplicons were purified using the sodium acetate ethanol method (Thermo Fisher Scientific), and sequencing reactions were run on a 3500xL Genetic Analyzer (Applied Biosystems, USA) (Myers and Miller, 1988; Altschul et al., 1990; Karlin and Altschul, 1990).

#### Phylogenetic analysis

Sequencing files were edited using CHROMASLITE (version 1.5) and further analyzed by Basic Local Alignment Search Tool (BLAST) with the closest culture sequence retrieved from the National Centre for Biotechnology Information (NCBI) database. BLAST finds regions of local similarity between sequences (Altschul et al., 1990). The program compares nucleotide or protein sequences to sequence databases and calculates the statistical significance of matches. The BLAST algorithms are used to infer functional and evolutionary relationships between sequences, as well as help identify members of gene families. The process involves an (i) initial search to find potentially closely related type strain sequences using the BLASTN program (Altschul et al., 1990), followed by a (ii) pairwise alignment to calculate the sequence similarity values between the query sequence and the sequences identified in the initial search step (i) (States et al., 1991). Therefore, each isolate is reported with the first five to ten hits observed in the database. Further multiple sequence alignment and phylogenetic analysis are recommended for accurate species prediction and evolutionary relationship (Myers and Miller, 1988; Karlin and Altschul, 1990).

#### Optimization of fermentation conditions

##### Effect of incubation period

To investigate the impact of the incubation period on bacterial growth, the C82 isolate was inoculated into MRS broth and incubated for different durations ranging from 24 to 120 h at 37 °C, both under static conditions and shaking at 140 rpm (Leslie et al., 2021).

##### Effect of carbon sources

The impact of various carbon sources on bacterial growth was evaluated by adding1% of different carbon sources, such as sucrose, starch, maltose, lactose, dextrose, fructose, inulin, and fructo-oligosaccharides (FOS), individually, to MRS broth. Additionally, FOS and inulin were tested at concentrations ranging from 1 to 6%. The cultures were then incubated at 37 °C for 72 h with shaking at 140 rpm (Wang et al., 2023). A 1% (w/v) concentration of carbon source was chosen to ensure sufficient supply of fermentable substrate to support active metabolism and lactic acid production without causing substrate inhibition or osmotic stress. This carbon-rich condition also promotes the synthesis of secondary metabolites, such as bacteriocins, rather than excessive biomass accumulation (Abedi and Hashemi, 2020).

##### Effect of nitrogen sources

To investigate the influence of different nitrogen sources on bacterial growth, C82 was cultured in MRS broth supplemented with 0.1% of various nitrogen sources including ammonium sulfate, ammonium carbonate, ammonium ferrous sulfate, tryptophan, peptone, and a mixture of branched-chain amino acids (leucine, valine, and isoleucine in a 1:1:1 ratio). The cultures were then incubated for 72 h at 37 °C (Wang et al., 2023). The concentration of 0.1% (w/v) nitrogen source was selected to maintain an optimal carbon-to-nitrogen balance for bacteriocin biosynthesis. A limited nitrogen level prevents excessive cell growth and promotes the transition into the stationary phase, during which bacteriocin production is typically enhanced. Additionally, a lower nitrogen concentration minimizes background protein interference during protein estimation and bioactivity assays, improving detection sensitivity (Krysenko, 2023).

##### Effect of pH

To evaluate the isolate’s pH tolerance, C82 was inoculated into MRS broth adjusted to pH values of 2, 4, 6, 8, and 10. The cultures were then incubated at 37 °C, and bacterial growth was monitored to assess pH-dependent viability (Indriati et al., 2014; Dwi Ludfiani et al., 2021).

##### Effect of bile salt

Bile salt tolerance was examined by culturing C82 in MRS broth supplemented with varying concentrations of bile salts ranging from 0.1 to 0.6%. The cultures were incubated at 37 °C, and growth was monitored to evaluate the strain’s ability to survive under bile stress conditions (Dwi Ludfiani et al., 2021; Upadhyay et al., 2025).

#### Bacteriocin production

To investigate bacteriocin production under optimized fermentation conditions, in selected isolates (C82), sterile liquid MRS broth was inoculated with a 72-h-old LAB culture of the selected isolate (10^8^ CFU/mL) and then incubated at the optimized fermentation conditions. The cultures were then subsequently centrifuged at 5000 rpm for 30 min, and the resulting cell-free supernatant was utilized as a crude bacteriocin extract for further analysis (Dates et al., 2025). Additionally, EPS production for the isolate was assessed by culturing the isolate on MRS agar supplemented with 1% sucrose and 1 mL of skim milk, followed by incubation at 37 °C for 48 h. Colonies exhibiting a shiny, ropy appearance were identified as positive for EPS production (Rühmann et al., 2015; Jurášková et al., 2025).

#### Evaluation of bacteriocin containing cell-free extract activity

##### Effect of temperature

To assess the effect of temperature, 1 mL of bacteriocin-containing cell-free extract was placed in a water bath at various temperatures, (20 °C, 40 °C, 60 °C, 80 °C, 100 °C, and 120 °C) for 30 min. After heat treatment, the samples were promptly cooled on ice. Antibacterial activity was determined by measuring the zone of inhibition against test organisms, and comparing the treated samples to untreated controls (Zangeneh et al., 2020).

##### Effect of bile salts

To explore the impact of bile salts, 1 mL of bacteriocin-containing cell-free extract was mixed with 1 mL of bile salt solutions at concentrations ranging from 0.1 to 0.6%, prepared in sterile distilled water. The mixtures were incubated at 37 °C for 30 min. Antibacterial activity was assessed using the agar well diffusion method and compared to untreated controls (Hassan et al., 2020).

##### UV radiation sensitivity

To investigate UV sensitivity, 1 mL of cell-free bacteriocin extract was exposed to UV light at various wavelengths for 15, 30, 45, and 60 min. Following exposure, antibacterial activity was tested against indicator strains using the agar well diffusion method, and the results were compared with non-irradiated controls (Otunba et al., 2022).

##### Bacteriocin stability assay

To evaluate the stability of the bacteriocin produced by *L. pentosus* C82, a bacteriocin stability assay was conducted. The crude bacteriocin extract was mixed with 0.1 mM buffer solutions adjusted to a pH range of 2–11 in a 1:1 (v/v) ratio. The mixtures were incubated at room temperature (37 °C) for 6–12 h and simultaneously at 4 °C for 6–12 h to assess stability under ambient and cold conditions. After incubation, the residual antibacterial activity of the bacteriocin was determined using the agar well diffusion method against *E. coli* and *S. marcescens* spp. LB agar plates were seeded with the indicator strains, and wells were filled with 50–100 μL of the bacteriocin-buffer mixtures. The plates were incubated at 37 °C for 18–24 h, and the zones of inhibition were measured. The antibacterial activity of the treated bacteriocin was compared to that of the untreated bacteriocin to assess stability across different pH values and temperatures. All experiments were conducted in triplicate for reproducibility, and the results were expressed as the percentage of residual antibacterial activity relative to the control (Abanoz and Kunduhoglu, 2018).

##### Effect of metal ions

The impact of metal ions on bacteriocin-containing cell-free extract was studied by adding monovalent and divalent metal salts, including Cu^2+^, Ca^2+^, Fe^3+^, Mg^2+^, Zn^2+^, K^+^, and Mn^2+^ at a final concentration of 0.1 mM. The extract was then incubated at 37 °C for 30 min. Antibacterial activity was assessed using the agar well diffusion method and compared to untreated controls (Abanoz and Kunduhoglu, 2018).

##### Effect of organic solvents and chemical agents on bacteriocin stability

To evaluate the stability of bacteriocin-containing cell-free extract in the presence of organic solvents and chemical agents, the C82 bacteriocin extract was treated with various compounds, including methanol, acetone, phenol, isopropanol (IPA), SDS, ethyl acetate, formaldehyde, chloroform, glycerol, hydrogen peroxide (H₂O₂), and Tween. Treated samples were then tested for antibacterial activity using the agar well diffusion method, and results were compared with untreated controls (Otunba et al., 2022).

#### Partial purification of bacteriocin

CFS from *Lactiplantibacillus pentosus* C82 cultures was obtained by centrifugation at 10,000 × g for 20 min at 4 °C. To precipitate bacteriocin-associated proteins, ammonium sulfate was slowly added to the chilled CFS under continuous stirring to achieve 60–80% saturation. The mixture was stirred for 1 h at 4 °C and incubated overnight at 4 °C to complete precipitation. The precipitated proteins were recovered by centrifugation at 10,000 × g for 30 min at 4 °C, and the resulting pellets were gently resuspended in 50 mM sodium phosphate buffer (pH 6.5). Desalting was performed 24 h by dialysis against 2 L of 0.01 M phosphate buffer (pH 7.0) at 4 °C, with regular buffer changes over 12 h. Total protein concentrations in the crude CFS and dialysed samples were quantified using the Bradford assay, with bovine serum albumin (BSA) used to generate the standard calibration curve. The molecular weight of the partially purified bacteriocin was estimated using 12% SDS-PAGE. Electrophoresis was performed at 150 V until the tracking dye reached the bottom of the gel. Following electrophoresis, gels were stained with Coomassie Brilliant Blue R-250, and destained for 24 h using a standard destaining solution containing acetic acid, methanol, and water. Molecular weight determination was carried out by comparing the migration of sample bands with pre-stained protein molecular weight markers (Goyal et al., 2018; Yuliana et al., 2023).

#### In-gel activity assay

The antimicrobial peptide produced by *Lactiplantibacillus pentosus* C82 was analyzed using an in-gel activity assay following SDS-PAGE. A 12% polyacrylamide resolving gel was prepared to enable the separation of molecular-weight bacteriocin peptides, and purified, concentrated C82 bacteriocin samples were loaded in duplicate. After electrophoresis, one portion of the gel containing molecular weight standards was stained with Coomassie Brilliant Blue R-250 to confirm peptide separation. The remaining unstained gel was processed for *in situ* antimicrobial activity detection. The gel was fixed in a mixture of 2-propanol, acetic acid, and distilled water (25:10:65, v/v/v) for 15 min and subsequently washed repeatedly with sterile distilled water for 30 min to remove SDS residues that could inhibit microbial growth. The gel was transferred to a sterile Petri dish and overlaid with 10 mL of 0.8% soft agar inoculated with *E. coli* and Serratia. After solidification, plates were incubated at 37 °C, and zones of inhibition corresponding to active bacteriocin bands were recorded, confirming the molecular weight-associated antimicrobial activity of the C82 bacteriocin (Baindara et al., 2013; Sharma et al., 2018).

#### LC–MS/MS analysis of partially purified bacteriocin

The molecular mass of the partially purified *L. pentosus* C82 bacteriocin was determined using liquid chromatography–tandem mass spectrometry (LC–MS/MS) following the method of Hu et al. (2017). The dialyzed protein fraction was reduced with 10 mM dithiothreitol (DTT) at 56 °C for 45 min and alkylated with 20 mM iodoacetamide (IAA) in the dark for 30 min. The sample was digested overnight at 37 °C using sequencing-grade modified trypsin (1:50, w/w, enzyme-to-substrate ratio) and desalted using C18 StageTips before MS analysis. Peptide mixtures were first loaded onto a Zorbax 300SB-C18 trap column (Agilent Technologies) and subsequently separated on a 0.15 mm × 150 mm RP-C18 analytical column using a linear gradient of 0.1% formic acid in acetonitrile at a flow rate of 300 nL/min. Mass spectrometric analysis was performed on a Q Exactive HF-X Orbitrap mass spectrometer (Thermo Fisher Scientific) operated in data-dependent acquisition (DDA) mode. Full MS scans (m/z 300–1800) were acquired at a resolution of 60,000, followed by MS/MS fragmentation of the top 10 most intense precursor ions using higher-energy collisional dissociation (HCD) (Xue et al., 2024).

#### Antioxidant activity

##### DPPH radical-scavenging activity

The DPPH radical-scavenging assay was performed with slight modifications. Bacteriocin from *L. pentosus* C82 at varying concentrations (20, 40, 60, 80, and 100 μg/mL) was mixed with a 0.1 mM DPPH methanolic solution and incubated in the dark for 30 min. The absorbance was then measured at 517 nm (Wang et al., 2023).


$$\begin{array}{l} \text{DPPH radical}-\text{scavenging activity}\;(\%)=\\ \;\;\;\;\;\;\;\;\;(A_{\text{control}}+A_{\text{blank}}-A_{\text{sample}})/A_{\text{control}}*\;100 \end{array}$$

##### ABTS radical-scavenging activity

The ABTS radical-scavenging capacity of C82 bacteriocin was assessed using a modified method based on Wang et al. (2023). The ABTS radical was created by mixing equal parts of 7 mM ABTS solution and 2.4 mM potassium persulfate, then left to incubate for 16 h at room temperature in the dark. Subsequently, 500 μL of bacteriocin solutions at various concentrations (20, 40, 60, 80, and 100 μg/mL) were mixed with 1 mL of ABTS radical solution and incubated in the dark at room temperature for 10 min. The absorbance was measured at 734 nm (Wang et al., 2023).

### *In vivo* developmental toxicity studies

#### Housing and maintenance of zebrafish (*Danio rerio*) and embryo collection

Zebrafish (*Danio rerio*) were obtained from a fish farm in Manimangalam, Chennai, at latitudes N 12° 55′1″ and longitude E 80° 2 29″. The fish were kept in 19-liter transparent aquaria maintained at 28.5 ± 0.5 °C with a 14:10 h light–dark cycle. They were fed live *Artemia salina* (brine shrimp) three times a day. After a 20-day acclimatization period, breeding was initiated using two mating pairs, each consisting of one male and one female. The breeding tanks had mesh bottoms to prevent egg predation. Embryos were collected 30 min after light onset, rinsed with freshly prepared E3 embryo medium, and incubated at 26 ± 1 °C until further experimentation, following OECD guidelines (OECD, 2013, 2019).

#### Determination of bacteriocin toxicity in the zebrafish model

Four hours post-fertilization, zebrafish embryos were placed into 12-well plates, with 10 embryos in each well (*n* = 30/group). The embryos were exposed to varying concentrations of bacteriocin (5, 10, 15, 20, 25, and 30 μg/mL), replenished every 24 h throughout the study. Each treatment group was conducted in triplicate. Embryonic development and morphological changes were observed using a stereo microscope at 4 × magnification (Umapathy and Pan, 2025a).

#### Therapeutic potential of bacteriocin in a gut dysbiosis-induced zebrafish model

To induce gut dysbiosis, zebrafish larvae were challenged with pathogenic bacterial strains, including *E. coli* and *S. marcescens*. Bacterial cultures were added to the water containing 5-day post-fertilization (dpf) zebrafish larvae. The infection was carried out for 48 h. Following dysbiosis induction, the larvae were treated with bacteriocin at optimized concentrations (determined from the toxicity assay) for 3–5 days. Gut dysbiosis induction was performed using serial dilutions of the bacterial cultures (10^−3^ to 10^−7^). For *E. coli*, only the 10^−3^ dilution was suitable for inducing gut dysbiosis without causing mortality; all other concentrations resulted in larval death. In the case of *S. marcescens*, gut dysbiosis was successfully induced at the 10^−4^ dilution, while all other concentrations were lethal to the larvae. This grouping and dilution selection allowed for the evaluation of bacteriocin’s therapeutic potential specifically against pathogen-induced gut dysbiosis under safe and stable conditions (Tan et al., 2019; Flores et al., 2023).

### *In vivo* antioxidant studies

#### Experimental groups and treatment protocol

Zebrafish (*Danio rerio*) embryos were allocated into five experimental groups (*n* = 30 per group) and transferred into sterile 12-well plates, with 10 embryos per well. Group 1 served as the untreated control. Group 2 consisted of embryos with gut dysbiosis induced by *E. coli*, while Group 3 had embryos with gut dysbiosis induced by *S. marcescens*. Group 4 included embryos with *E. coli*-induced dysbiosis treated with bacteriocin, and Group 5 consisted of embryos with *S. marcescens*-induced dysbiosis treated with bacteriocin (20 μg/mL) derived from *Lactiplantibacillus pentosus* C82.

Bacteriocin treatment was administered via immersion for seven consecutive days following pathogen exposure. After the treatment period, embryos were pooled (n = 30 per group) and homogenized in chilled 100 mM Tris–HCl buffer (pH 7.8) containing 150 mM KCl and 1 mM EDTA. Homogenates were centrifuged at 10,000 rpm for 15 min at 4 °C, and the supernatants were collected for enzymatic antioxidant assays. Protein concentrations were determined using Bradford’s method. All biochemical analyses were performed in triplicate.

##### Superoxide dismutase (SOD) activity

A reaction mixture was prepared containing 50 mM phosphate buffer (pH 7.8), 100 μM EDTA, 750 μM NBT, 130 mM methionine, and 20 μM riboflavin. 50 μL of homogenate was added, and the mixture was exposed to light for 20 min. Absorbance was measured at 560 nm to determine SOD activity (Santhi et al., 2025).

##### Catalase (CAT) activity

For CAT estimation, 50 μL of homogenate was mixed with 100 μL of buffered hydrogen peroxide (H₂O₂). The degradation of H₂O₂ was measured at 240 nm at 15-s intervals over 2 min. Assays were performed in accordance with standard spectrophotometric protocols (Janarathanam et al., 2024).

##### Reduced glutathione (GSH) and glutathione S-transferase (GST) assays

The reaction mixture contained 20 mM DTNB, 10 μM GSH, 60 μM CDNB, and 150 μL of 100 mM phosphate buffer (pH 7.4). 50 μL of sample homogenate was added. Absorbance was recorded at 412 nm (for GSH) and 340 nm (for GST) to evaluate antioxidant status (Santhi et al., 2025).

##### Lipid peroxidation (LPO) assay

Malondialdehyde (MDA) levels were estimated as markers of lipid peroxidation. 0.1 mL of 5% trichloroacetic acid (TCA) was added to 100 μL of homogenate and incubated on ice for 15 min. 0.2 mL of 0.67% thiobarbituric acid (TBA) was added, followed by heating in a water bath at 100 °C for 30 min. After rapid cooling, samples were centrifuged at 2000 rpm for 10 min (4 °C). The absorbance of the supernatant was measured at 532 nm (Janarathanam et al., 2024).

##### Nitric oxide (NO) assay

NO levels were assessed using a modified Griess reaction. 100 μL of Griess reagent was mixed with the homogenized sample and incubated at room temperature for 25 min. The absorbance was then recorded at 540 nm (Umapathy and Pan, 2025b).

#### Gene expression study

RNA was extracted from adult zebrafish (*n* = 6 per group) using the RDP Trio™ Reagent. Gene-specific primers were designed using NCBI Primer-BLAST (Table 1). Quantitative gene expression analysis was performed with the AURA 2 × One-Step RT-PCR Master Mix. Reverse transcription was carried out at 44–50 °C for 15 min, followed by an initial denaturation and enzyme activation step at 95 °C for 3 min. The amplification protocol consisted of 40 cycles of denaturation at 95 °C for 10 s, annealing at 60 °C for 45 s, and extension at 72 °C for 15 s. Relative gene expression levels were determined using the comparative 2 − ΔΔCt method (Velumani et al., 2024).

**Table 1:** Primer sequence used in the real-time PCR analysis.

Sl. no.	Primer type	Forward	Reverse	Accession number
1.	IL10	CTTTAAAGCACTCCACAACCCCAA	CTTGCATTTCACCATATCCCGCTT	NM_001020785.2
2.	TNF-α	TAGAACAACCCAGCAAACTC	TCTCCTTCTTCAACATCCAA	NM_212859.2
3.	Claudin 5a	GGTCATCTCCTCGGTCTTGA	GCACCTGCGGGTTATAGAAG	NM_213274.1
4.	ZO-1	CACGAGACAAACTGGCAAGA	TCCAGCACTGCATGCTTATC	BI706952.1
5.	All Bifidobacteria	GGGATGCTGGTGTGGAAGAGA	TGCTCGCGTCCACTATCCAGT	DQ298393.1
6.	All Lactobacillus spp.	TGGATGCCTTGGCACTAGGA	AAATCTCCGGATCAAAGCTTACTTAT	AY365115.1
7.	All Enterococcus spp.	AGAAATTCCAAACGAACTTG	CAGTGCTCTACCTCCATCATT	HM007611.1 NM_131327.1
8.	*csgD*	TGATGAACAACGAACGAGCGATCTC	GCTTGCCAGTTACCTGATTACACATTC	PV012319.1
9.	*bsmA*	TAGTCCGCACACTCATCGC	GATCTCCTGCGCCTGTGC	AF537272.1
10.	Nfe2l2a	GGCGATCCTCCTGTAAACCC	CGAAGGATCCGTCTTCGGTT	NM_182889.1
11.	Hmox1a	GCTTCTGCTGTGCTCTCTATACG	CAATCTCTCTCAGTCTCTGTGC	NM_001127516.1
12.	Beta-actin	AAGCTGTGACCCACCTCACG	GGCTTTGCACATACCGGAGC	NM_131031.2

#### Statistical analysis

Each experiment in this study was conducted in triplicate, and the results are presented as mean ± SD. The uncertainty was reported by implementing the Thumb rule. GraphPad Prism 5.0 (GraphPad Software, Inc., San Diego, CA) was used to analyze the data using the Tukey Multiple Comparison Test and one-way analysis of variance (ANOVA). The data were indicated with “*” and were considered significant when the *p*-value was less than 0.05 (Priyanath et al., 2020).

## Results

### Isolation of LAB from a nonconventional source

**Figure 1:**
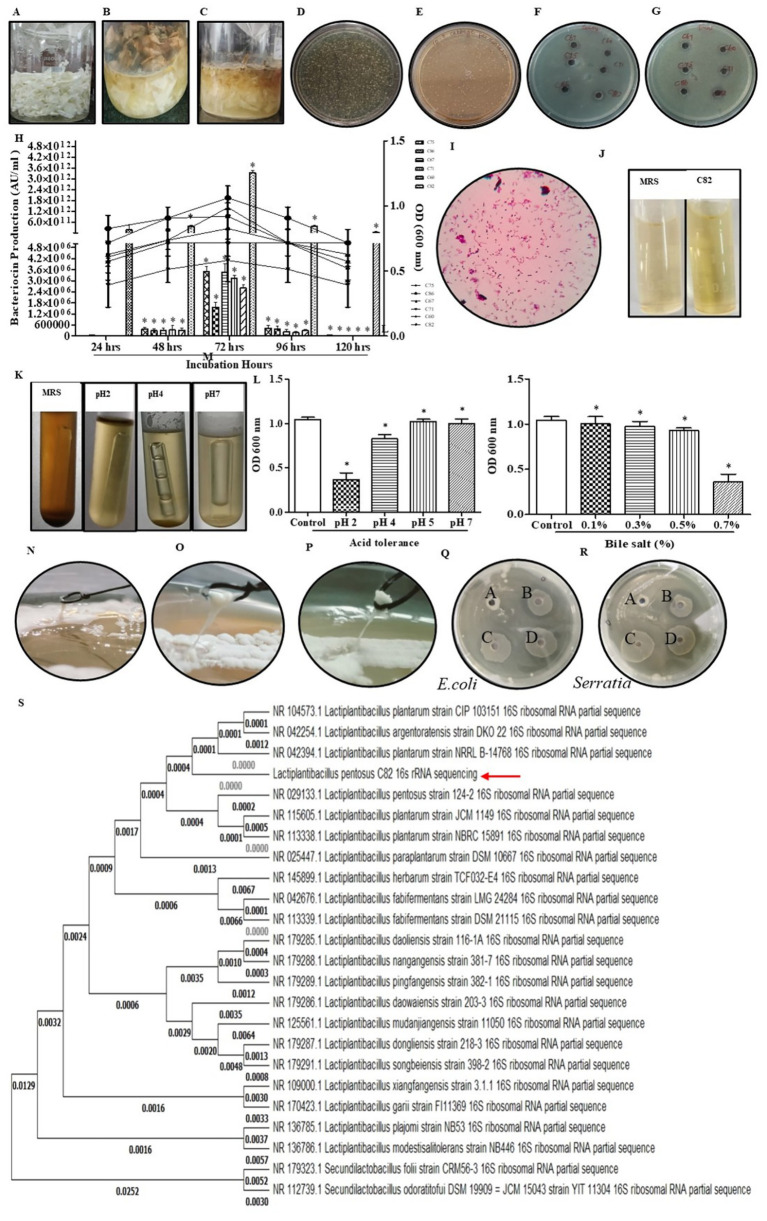
Screening and identification of bacteriocin-producing *Lactiplantibacillus pentosus* C82 isolated from sauerkraut. **(A–C)** Sauerkraut fermentation showing microbial growth progression from day 0 to day 35 with MRS agar plate counts at 10^−3^ dilution. **(D)** Bacterial load observed in MRS at 10^−3^ dilution, and **(E)** bacterial load observed in nutrient agar at 10^−3^ dilution. **(F,G)** Antimicrobial activity of bacteriocin against *E. coli* and *S. marcescens*. **(H)** Screening of isolate C82 for bacteriocin production. **(I)** Gram staining and microscopic observation confirming Gram-positive rod morphology. **(J)** Confirmation of lactic acid bacteria (LAB) using the ferric chloride test. **(K)** Fermentation and gas detection in MRS under different pH conditions (pH 2–7). **(L)** Acid tolerance assay at different pH values (2–7). **(M)** Bile salt tolerance assay at concentrations ranging from 0.1–0.7%. **(N–P)** EPS production by isolate C82 in MRS and MRS supplemented with skim milk. **(Q,R)** Evaluation of antimicrobial activity of *Lactiplantibacillus pentosus* C82 cell-free supernatant (CFS) after pH neutralization and catalase treatment. **(S)** Phylogenetic tree for isolate C82, identified as *Lactiplantibacillus pentosus* C82. The data were considered significant (*p* < 0.05) and marked by the symbol “*”.

After a 35-day fermentation of *Brassica oleracea* var. *capitata*, approximately 2.5 × 10^8^ CFU/mL of colonies were isolated, with 2.1 × 10^8^ CFU/mL, and 90 strains identified as LAB, as depicted in Figures 1A–E. Out of the isolated strains, 6 isolates (C60, C67, C71, C75, C82, and C86) exhibited antibacterial activity against *E. coli* and *S. marcescens*. Notably, isolate C82 displayed the highest antibacterial activity, with 1.5 cm and 1.3 cm inhibition zones against *E. coli* and *S. marcescens,* respectively, as illustrated in Figures 1F–G. In addition to its potent antibacterial activity, isolate C82 also demonstrated superior bacteriocin production compared to the other isolates shown in Figure 1H, indicating its potential as a promising LAB candidate. Further Gram staining confirmed that the isolates were Gram-positive, non-spore-forming bacilli, which are key characteristics of LAB as shown in Figure 1I.

#### Morphological and biochemical characterization of LAB isolates

The isolate C82 with the highest antibacterial activity and bacteriocin production was selected for further morphological and biochemical characterization. Catalase activity testing revealed that strain C82 is catalase-negative, and the gas production test confirmed the isolate as heterofermentative (Figures 1J,K). It also exhibited efficient fermentation of glucose, lactose, sucrose, and galactose, as indicated by a red-to-yellow colour change. The presence of lactic acid as a fermentation product was confirmed by a light yellow-green color change. Additionally, the growth analysis of the isolate showed its tolerance to acidic conditions (pH 2), with significant growth at pH 5, as shown in Figure 1L. In the bile tolerance assay, C82 demonstrated 88% survivability in MRS broth containing 0.5% bile salts after 24 h, as depicted in Figure 1M.

Furthermore, EPS production of C82 was confirmed by its shiny, mucoid, and slightly ropy textures, with increased ropiness upon the addition of skim milk. Unlike typical EPS-overproducing strains, C82’s ropy phenotype coincided with high bacteriocin activity, suggesting a synergistic contribution of both metabolites. Microscopic examination confirmed that isolate C82 is a rod-shaped, gram-positive, non-spore-forming bacterium, measuring 5.3–5.5 μm in length and 1.3–1.5 μm in width (Figures 1N–P).

Neutralizing the CFS to pH 6.5 completely eliminated antimicrobial activity, as shown in the neutralized sample (Figures 1Q,R). The neutralized sample (C) produced no-inhibition zone against *E. coli* and *S. marcescens*, in contrast to the untreated CFS (B), which displayed clear and strong inhibition zones. The absence of inhibition in the neutralized CFS confirms that organic acids, particularly lactic acid, play a significant role in the antimicrobial effect. However, catalase-treated CFS (D) exhibited inhibition zones similar to the untreated CFS (B), indicating that hydrogen peroxide does not contribute to the antimicrobial activity of C82. The buffer control (A) showed no inhibition, confirming the specificity of the assay. This suggests hydrogen peroxide is not involved in the antimicrobial effect of C82. The consistent inhibitory activity following catalase addition confirms that the active compound is not an oxidative metabolite but rather a catalase-resistant, protein-based bacteriocin.

#### Identification of selected isolate C82

Molecular identification through 16S rRNA gene sequencing confirmed that C82 shares 99.76% sequence similarity with *Lactiplantibacillus pentosus* (Figure 1S). It also exhibited close homology to *Lactiplantibacillus plantarum* (99.67%) and *Lactiplantibacillus paraplantarum* (99.73%). Phylogenetic analysis using the Maximum Likelihood method placed C82 in close evolutionary proximity to *Lactiplantibacillus pentosus* strain NR_029133.1. The 16S rRNA sequence of isolate C82 has been submitted to GenBank under the accession number [PP860573].

### Optimization of bacteriocin production by *Lactiplantibacillus pentosus* C82

To optimize bacteriocin production by *L. pentosus* C82, various physiological and nutritional factors were investigated. Bacteriocin activity was quantified as arbitrary units per milliliter (AU/mL), and cell growth was monitored via optical density at 600 nm (OD₆₀₀).

#### Effect of incubation period

**Figure 2:**
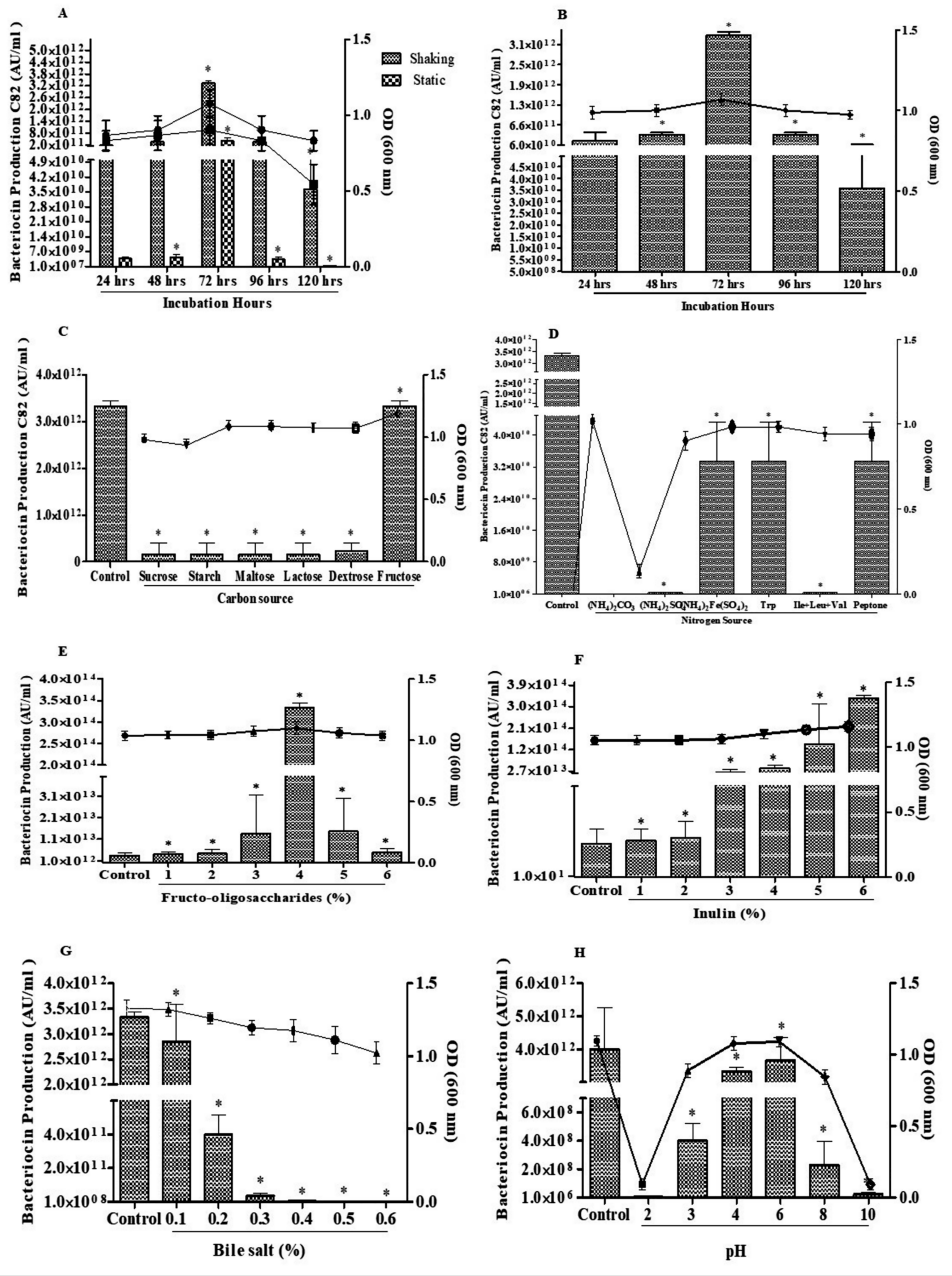
Optimization of growth conditions for bacteriocin production by *Lactiplantibacillus pentosus* C82. **(A)** Effect of incubation time under shaking and static conditions. **(B)** Influence of incubation hours on bacteriocin yield. **(C)** Effect of different carbon sources. **(D)** Effect of nitrogen sources. **(E)** Effect of different concentrations of fructo-oligosaccharides (1–6%). **(F)** Effect of inulin concentrations (1–6%). **(G)** Effect of bile salt concentrations (0.1–0.7%). **(H)** Effect of pH (2–10). The data were considered significant (*p* < 0.05) and marked by the symbol “*”.

Bacteriocin production by isolate C82 was significantly influenced by the incubation period under both shaking and static conditions. As illustrated in Figures 2A,B, production increased steadily starting at 24 h and peaked at 72 h under shaking conditions. This peak coincide with the highest OD and demonstrated strong antibacterial activity. However, beyond this point (96–120 h), bacteriocin levels increased significantly. On the other hand, static cultures consistently showed lower bacteriocin yields, highlighting the crucial role of aeration in promoting enhanced bacteriocin production.

#### Effect of carbon sources

The influence of different carbon sources on bacteriocin production by *L. pentosus* C82 was evaluated (Figure 2C). Among the tested carbohydrates, fructose significantly enhanced bacteriocin production levels compared to the control (3.22 × 10^12^ AU/mL). In contrast, sucrose, starch, maltose, lactose, and dextrose exhibited only minimal bacteriocin production, less than 0.5 × 10^12^ AU/mL (*p* < 0.05).

#### Effect of nitrogen sources

The effect of various nitrogen sources on bacteriocin production by *L. pentosus* C82 is presented in Figure 2D. The control medium supported the highest bacteriocin production (3.22 × 10^12^ AU/mL). Supplementation with organic nitrogen sources such as tryptophan, peptone, and the amino acid mixture (Ile-Leu-Val) resulted in moderate bacteriocin yields (1.5–2.0 × 10^12^ AU/mL), significantly lower than the control (p < 0.05). In contrast, inorganic nitrogen sources, including ammonium carbonate and ammonium sulfate, suppressed bacteriocin production levels (<0.5 × 10^12^ AU/mL).

#### Effect of fructo-oligosaccharides (FOS)

The impact of various concentrations of FOS and inulin on bacteriocin production by *L. pentosus* C82 is illustrated in Figures 2E,F. Among the tested concentrations (1–6%) of FOS supplementation, 4% FOS significantly increased bacteriocin production to 3.5 × 10^14^ AU/mL. Moderate bacteriocin production was observed at 3 and 5% FOS (1.0–1.5 × 10^13^ AU/mL), while lower (1–2%) and higher (6%) concentrations only minimally supported production. Conversely, supplementation with 1 and 2% inulin resulted in a slight increase (3.22 × 10^12^ AU/mL), showing no significant improvement over the control. However, with 3 and 4% inulin, bacteriocin production reached 3.33 × 10^13^ AU/mL. The maximum production was observed at 5 and 6% inulin, yielding 3.33 × 10^14^ AU/mL. Collectively, these findings suggest that inulin supplementation promotes bacteriocin production by *L. pentosus* C82 in a dose-dependent manner, while FOS supplementation enhanced production at 4%.

#### Effect of bile salt concentration

The impact of different concentrations of bile salt on bacteriocin production by *L. pentosus* C82 is depicted in Figure 2G. The control medium supported the highest bacteriocin production (3.22 × 10^12^ AU/mL). At 0.1% bile salt, bacteriocin production remained unchanged compared to the control. However, decreased production was observed at 0.2 and 0.3%, ranging from 3.33 × 10^11^ to 3.33 × 10^10^ AU/mL, respectively. At higher bile salt concentrations (0.4–0.6%), bacteriocin production was significantly inhibited, with levels reduced to the range of 10^7^–10^9^ AU/mL. Bile salt strongly inhibited both bacteriocin production and bacterial growth in *L. pentosus* C82, with concentrations above 0.2% causing a suppression of bacteriocin production.

#### Effect of pH

The impact of different pH levels on bacteriocin production by C82 is illustrated in Figure 2H. The control medium supported high bacteriocin production (3.22 × 10^12^ AU/mL). At pH 2, and 3 bacteriocin production decreased to 2.33 × 10^6^ AU/mL and 3.33 × 10^8^ AU/mL, respectively. A significant increase was observed at pH 4 and 6, where bacteriocin yields (3.22 × 10^12^ AU/mL) were comparable to the control, indicating optimal production under near-neutral conditions. However, production declined again at pH 8 (3.33 × 10^8^ AU/mL) and was almost completely suppressed at pH 10 (3.33 × 10^7^ AU/mL). Bacteriocin production and bacterial growth by *L. pentosus* C82 were optimal at pH 4–6, as shown in Figure 2H.

### Activity of bacteriocin produced by *Lactiplantibacillus pentosus* C82

Under the optimized fermentation conditions, with a 72-h incubation period under shaking, fructose as the carbon source, peptone as the nitrogen source, supplemented with 4% FOS and 6% inulin, *L. pentosus* C82 exhibited maximum bacteriocin production. This optimized combination significantly enhanced bacteriocin yield compared to individual parameters, indicating a synergistic effect of prebiotic supplementation on bacteriocin biosynthesis.

#### Effect of temperature on bacteriocin activity

**Figure 3:**
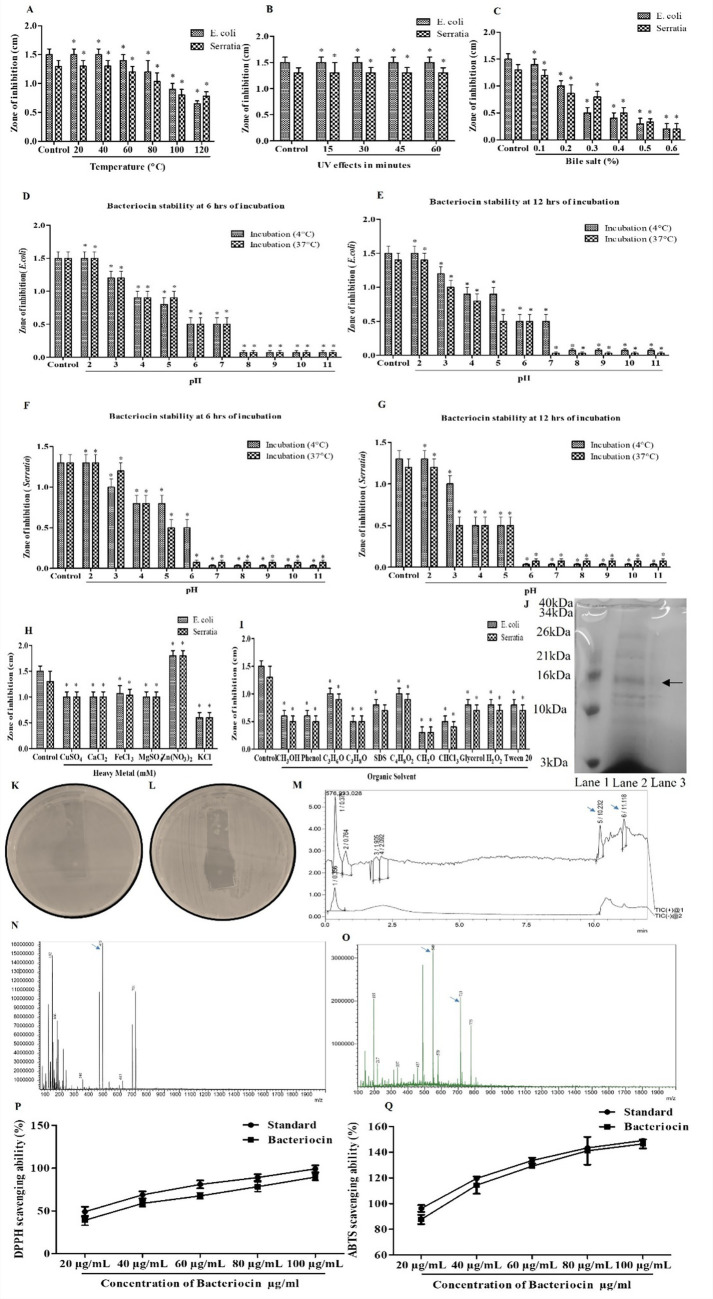
Stability and functional characterization of bacteriocin produced by *Lactiplantibacillus pentosus* C82. **(A)** Effect of temperature. **(B)** Effect of UV exposure. **(C)** Effect of bile salt concentrations. **(D–G)** pH stability assays at 6 h and 12 h incubation showing bacteriocin activity against *E. coli* **(D,E)** and *S. marcescens* **(F,G)**. **(H)** Effect of heavy metal ions. **(I)** Effect of organic solvents. **(J)** SDS-PAGE showing lane 1: protein molecular weight marker (3–40 kDa), lane 2: dialyzed bacteriocin sample, and lane 3: crude CFS of C82. **(K,L)** In-gel activation assay demonstrating the antimicrobial activity of the excised 16 kDa protein band against indicator pathogens *E. coli* **(K)** and *S. marcescens* **(L)**. **(M–O)** LC–MS/MS analysis of the active antimicrobial fraction, with blue arrows indicating the major chromatographic peaks (RT 10.232–11.118 min) and key fragment ions (*m/z* 475, 549, 713) associated with the peptide. **(N,O)** Antioxidant potential of bacteriocin showing DPPH **(P)** and ABTS **(Q)** radical scavenging activity. The data were considered significant (*p* < 0.05) and marked by the symbol “*”.

Bacteriocin from C82 demonstrated temperature-dependent activity (Figure 3A). Maximum antibacterial activity was observed against both *E. coli* and *S. marcescens* at 20 °C and 40 °C, with a zone of inhibition of 1.5 cm and 1.3 cm, respectively. Bacteriocin activity was moderately reduced at 60–80 °C with a smaller zone of inhibition, and significantly inactivated at 100–120 °C against both test organisms.

#### Effect of UV radiation

Bacteriocin from *L. pentosus* C82 demonstrated UV stability (Figure 3B). Maximum antibacterial activity was observed against *E. coli* and *S. marcescens* in the control, with inhibition zones of 1.5 cm and 1.3 cm, respectively. Exposure to UV for 15, 30, 45, and 60 min did not cause any significant reduction in bacteriocin activity. The bacteriocin activity of *L. pentosus* C82 was stable under UV exposure up to 60 min, retaining its inhibitory effect against both test organisms, with a zone of inhibition of 1.5 cm for *E. coli* and 1.3 cm for *S. aureus.*

#### Effect of bile salt concentration

Bacteriocin from *L. pentosus* C82 demonstrated bile salt–dependent activity (Figure 3C). Maximum antibacterial activity was observed in the control, with inhibition zones of 1.5 cm against *E. coli* and 1.3 cm against *S. marcescens*. Activity remained stable at 0.1% bile salt (1.4 cm and 1.2 cm, respectively), but was sharply reduced from 0.2% onwards. Moderate inhibition was observed at 0.2–0.3%, while activity was almost completely reduced at 0.4–0.6%, with the lowest inhibition zone of 0.2 cm against both test organisms.

#### Bacteriocin stability assay

The stability of the bacteriocin produced by *L. pentosus* C82 was assessed across a pH range of 2–11, following incubation at 4 °C and 37 °C for 6 and 12 h, as shown in Figures 3D–G. The bacteriocin exhibited maximum stability under acidic conditions, retaining full activity at pH 2–3, with inhibition zones of 1.0–1.5 cm. Moderate activity was observed at mildly acidic conditions (pH 4–5; 0.5–0.9 cm), whereas reduced activity was observed under neutral and alkaline conditions (pH ≥ 6). Prolonged incubation (12 h) resulted in a slight reduction of activity at acidic pH, particularly at 37 °C, though the overall trend remained unchanged. Collectively, these findings indicate that C82 bacteriocin is highly stable and bioactive under acidic environments, moderately tolerant at mildly acidic pH, and rapidly inactivated under neutral to alkaline conditions, with stability better preserved at 4 °C than at 37 °C, suggesting cold storage enhances its functional integrity.

#### Effect of heavy metals

The effect of metal ions on the activity of the bacteriocin from *L. pentosus* C82 was evaluated as shown in Figure 3H. Compared with the control (1.5 cm and 1.3 cm, respectively), bacteriocin activity decreased in the presence of Cu^2+^, Ca^2+^, Mg^2+^, and Fe^3+^ (zone of inhibition- 1.0–1.06 cm), while K^+^ caused a significant reduction (0.6 cm). In contrast, Zn^2+^ markedly enhanced activity, yielding the largest inhibition zones (1.8 cm). The C82 bacteriocin displayed moderate tolerance to most divalent ions, while it was destabilized by K^+^, and significantly strengthened by Zn^2+^, underscoring the modulatory influence of metal ions on bacteriocin functionality.

#### Effect of organic solvents and chemical agents

The bacteriocin from *Lactiplantibacillus pentosus* C82 exhibited differential stability in the presence of organic solvents and chemical agents, as shown in Figure 3I. Formaldehyde caused the loss of activity (0.3 cm), followed by chloroform and propanol (0.4–0.5 cm). Methanol and phenol similarly reduced activity (0.5–0.6 cm), while SDS, glycerol, H₂O₂, and Tween 20 maintained moderate inhibition (0.7–0.8 cm). Acetone and butanol preserved comparatively higher activity (0.9–1.0 cm). Overall, the bacteriocin was highly sensitive to strong denaturants but showed moderate resilience in less aggressive solvents and surfactants.

### Partial purification of bacteriocin

The bacteriocin from *Lactiplantibacillus pentosus* C82 was recovered through ammonium sulfate precipitation at 70% saturation, followed by centrifugation to obtain the protein pellet. The pellet was gently dissolved in 0.01 M phosphate buffer (pH 7.0) and subsequently purified by dialysis against 0.01 M phosphate buffer (pH 7.0) to remove salts and low-molecular-weight impurities. The partially purified, dialyzed fraction exhibited a substantial improvement in purity, resulting in a 13.29-fold increase in specific activity compared to the crude extract. The bacteriocin preparation exhibited a specific activity of 700 AU/mg, with an overall activity yield of 54.68% and a total protein content of 10 mg retained after purification (Table 2).

**Table 2:** Purification of bacteriocin from *L. pentosus* C82.

Fraction	Volume (mL)	Total protein (mg)	Total activity (AU)	Specific activity (AU/mg)	Purification fold	Yield (%)
Crude CFS	100	243	12,800	52.67	1.0	100%
Protein precipitation	20	30	9,200	306.67	5.82	71.88%
Dialyzed fraction	15	10	7,000	700.00	13.29	54.68%

SDS-PAGE analysis of the dialyzed fraction revealed a single, well-defined protein band, indicating successful enrichment of the active component. Based on migration comparison with pre-stained molecular weight markers, the bacteriocin was estimated to have a molecular mass of 16 kDa (Figure 3J). To confirm that the band observed on SDS-PAGE corresponded to the antimicrobial component, an in-gel antimicrobial activity assay was conducted. The gel strip containing the 16 kDa band was carefully placed on agar plates that had been seeded with *E. coli* and *S. marcescens* (Figures 3K,L). A clear zone of inhibition was seen around the gel fragment on both plates, indicating that the 16 kDa protein band is indeed the bioactive bacteriocin produced by C82. These findings confirm that the partially purified, 16 kDa protein is responsible for the antimicrobial activity of *L. pentosus* C82, supporting its classification as a proteinaceous bacteriocin.

### LC–MS/MS analysis of partially purified bacteriocin

The partially purified antimicrobial fraction derived from *L. pentosus* C82 exhibited multiple compounds eluting in the early phase of the chromatogram. Two prominent late-eluting peaks at RT 10.232 min and 11.118 min (Figure 3M) represented the major hydrophobic species and corresponded to the fraction exhibiting antimicrobial activity in the in-gel overlay assay. LC–MS/MS analysis of this active fraction (RT 10.2–11.1 min) revealed peptide-like fragmentation patterns. In the first MS/MS event, a dominant base peak at m/z 475 was detected alongside major fragment ions at m/z 132, 166, 340, 457, 613, and 701. A second MS/MS event produced a complementary spectrum with a base peak at m/z 549 and additional intense ions at m/z 195, 217, 337, 457, 579, 713, and 775 (Figures 3N,O). The sequential high-intensity fragment ions observed across both spectra are characteristic of b- and y-type peptide backbone cleavages, confirming the proteinaceous nature of the bioactive compound. Collectively, the chromatographic retention profile, peptide-specific fragmentation signatures, and alignment with a 16 kDa band displaying antimicrobial activity strongly indicate that the active molecule produced by *L. pentosus* C82 is a peptide bacteriocin or bacteriocin-like inhibitory substance (BLIS).

### Antioxidant activity of bacteriocin derived from *Lactiplantibacillus pentosus* C82

The DPPH radical-scavenging assay was used to assess the antioxidant potential of bacteriocin produced by *L. pentosus* C82, with ascorbic acid used as the standard control. As depicted in Figure 3P, the radical-scavenging activity of bacteriocin increased in a concentration-dependent manner. In the DPPH assay, bacteriocin showed a concentration-dependent rise in free radical-scavenging activity, reaching 38.3, 58.3, 67, 78, and 89% inhibition at 20, 40, 60, 80, and 100 μg/mL, respectively. The IC₅₀ value of the bacteriocin for DPPH was calculated as 31.1 μg/mL, compared to 21.0 μg/mL for ascorbic acid. Similarly, in the ABTS assay, the bacteriocin exhibited strong antioxidant efficacy, with inhibition values of 87.7, 113.6, 128.6, 140.6, and 146% at the same respective concentrations as shown in Figure 3Q. The ABTS IC₅₀ of the bacteriocin was found to be 11.4 μg/mL, closely matching the standard ascorbic acid value of 10.4 μg/mL. These results suggest that bacteriocin from *L. pentosus* C82 possesses potent and dose-dependent radical-scavenging activity.

### *In vivo* developmental toxicity studies

**Figure 4:**
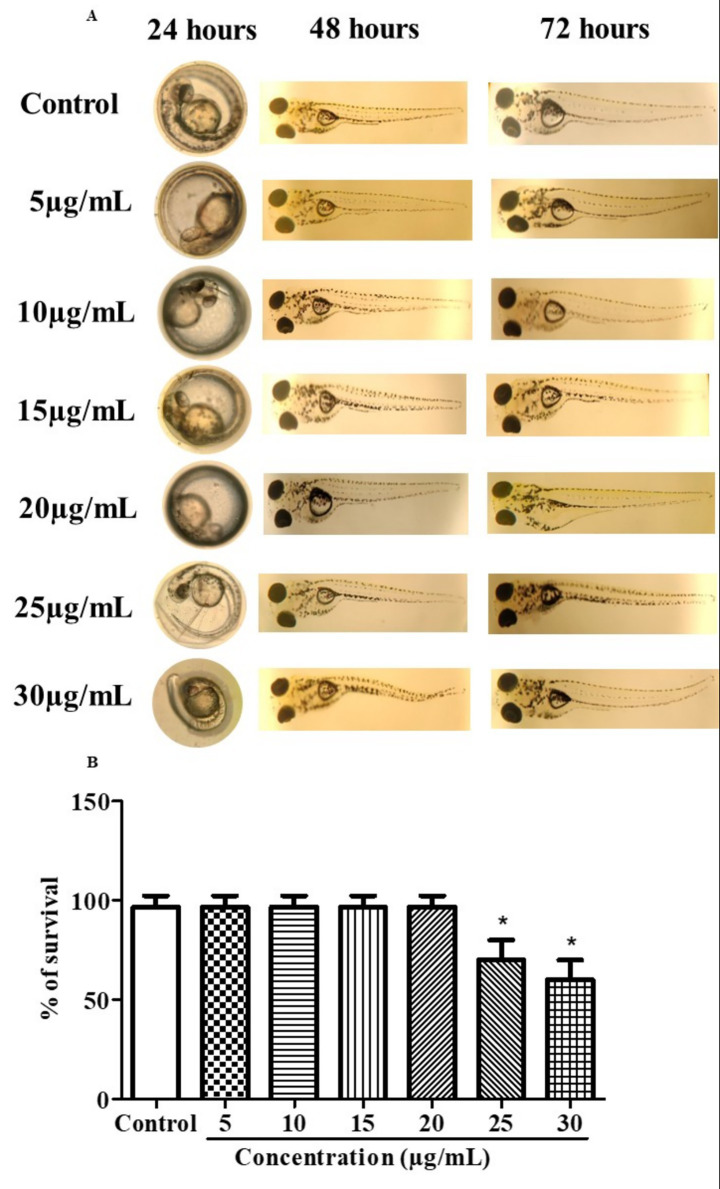
*In vivo* developmental toxicity. **(A)** Bacteriocin in zebrafish embryos and larvae. **(B)** Percentage of survival. The data were considered significant (*p* < 0.05) and marked by the symbol “*”.

*In vivo* developmental toxicity studies revealed that zebrafish embryos exposed to C82 bacteriocin at concentrations of 5, 10, 15, and 20 μg/mL did not show any significant morphological abnormalities compared to the control group. The embryos progressed normally over 24, 48, and 72 h, indicating a high tolerance to the bacteriocin at these concentrations (Figure 4A). However, at higher concentrations of 25 μg/mL and 30 μg/mL, embryos displayed mild developmental changes, such as delayed hatching and slight pericardial edema. These changes were linked to a noticeable decrease in survival rate, which decreased to approximately 65% at 25 μg/mL and 55% at 30 μg/mL compared to the control (Figure 4B). In summary, these findings suggest that C82 bacteriocin is safe and non-toxic at concentrations up to 20 μg/mL, while higher concentrations (>25 μg/mL) lead to dose-dependent developmental toxicity in zebrafish embryos.

### Therapeutic potential of bacteriocin in a zebrafish gut dysbiosis model

Zebrafish larvae were divided into five groups to evaluate treatment effects. Group 1 served as the untreated control, while Group 2 was induced with gut dysbiosis using *E. coli*, and Group 3 was induced with gut dysbiosis using *S. marcescens*. Group 4 induced with *E. coli*-induced dysbiosis was treated with bacteriocin (20 μg/mL), whereas Group 5 induced with *S. marcescens*-induced dysbiosis was also treated with bacteriocin (20 μg/mL). This grouping enabled the evaluation of bacteriocin’s therapeutic potential against pathogen-induced gut dysbiosis.

### *In vivo* antioxidant activity

#### SOD assay

**Figure 5:**
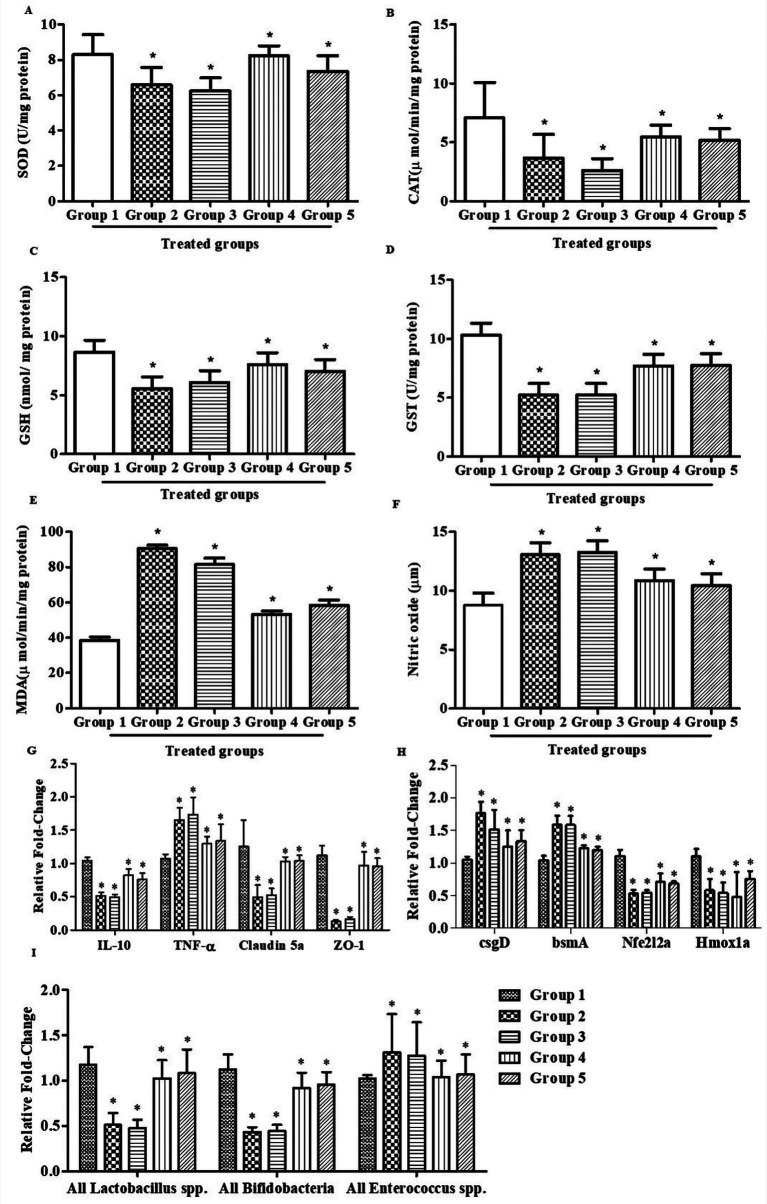
Effect of C82 bacteriocin treatment on oxidative stress biomarkers, cytokine expression, and gut microbial gene modulation in zebrafish larvae. **(A–F)** Antioxidant enzyme and oxidative stress marker activities across treatment groups: **(A)** Superoxide dismutase (SOD), **(B)** catalase (CAT), **(C)** reduced glutathione (GSH), **(D)** glutathione *S*-transferase (GST), **(E)** malondialdehyde (MDA), and **(F)** nitric oxide (NO). **(G)** Relative mRNA expression of IL-10, TNF-*α*, Claudin 5a, ZO-1. **(H)** Expression levels of oxidative stress-related (Nfe2l2a, Hmox1a) and bacterial biofilm-associated genes (*csgD, bsmA*). **(I)** Relative abundance of gut microbiota-related genes (*Lactobacillus* spp.*, Bifidobacteria*, and *Enterococcus* spp.). The data were considered significant (*p* < 0.05) and marked by the symbol “*”.

The superoxide dismutase activity exhibited notable variations across groups, as shown in Figure 5A. Group 1 showed the highest SOD activity (8.30 U/mg protein). Dysbiosis-induced Group 2 and 3 significantly reduced SOD levels to 6.58 U/mg protein (20.7% decrease) and 6.23 U/mg protein (24.9% decrease), respectively. Remarkably, bacteriocin treatment restored antioxidant activity, with Groups 4 and 5 showing 8.23 U/mg protein (0.8% reduction) and 7.34 U/mg protein (11.5% reduction), nearly equivalent to the control. These findings indicate that bacteriocin supplementation effectively alleviates oxidative stress by restoring SOD activity in gut dysbiosis models.

#### CAT assay

Catalase activity exhibited significant alterations across the treatment groups, as shown in Figure 5B. Group 1 showed the highest CAT activity (7.09 μmol/mg protein). Gut dysbiosis induced by *E. coli* in Group 2 markedly reduced CAT levels to 3.68 μmol/mg protein (48.0% decrease), while *S. marcescens*-induced dysbiosis in Group 3 further decreased activity to 2.61 μmol/mg protein (63.2% decrease). Bacteriocin supplementation significantly restored CAT activity, with Group 4 reaching 5.46 μmol/mg protein (22.9% reduction) and Group 5 showing improvement to 5.17 μmol/mg protein (27.0% reduction). These findings indicate that bacteriocin treatment effectively mitigates oxidative stress by restoring catalase activity in dysbiosis-induced models.

#### GSH estimation

GSH activity exhibited significant changes across the treatment groups, as shown in Figure 5C. Group 1 showed the highest GSH activity (8.65 μmol/mg protein). Gut dysbiosis induced by *E. coli* in Group 2 markedly reduced GSH levels to 5.55 μmol/mg protein (35.8% decrease), while *S. marcescens*-induced dysbiosis in Group 3 decreased activity to 6.08 μmol/mg protein (29.7% decrease). Bacteriocin supplementation significantly restored GSH activity, with Group 4 reaching 7.61 μmol/mg protein (12.0% reduction compared to control) and Group 5 showing recovery to 7.02 μmol/mg protein (18.9% reduction). These findings suggest that bacteriocin treatment effectively enhances redox balance by restoring GSH levels in dysbiosis-induced models.

#### GST estimation

GST activity exhibited marked variations across the treatment groups, as shown in Figure 5D. Group 1 showed the highest GST activity (10.33 U/mg protein). Gut dysbiosis induced by *E. coli* in Group 2 sharply reduced GST levels to 5.21 U/mg protein (49.5% decrease), while *S. marcescens*-induced dysbiosis in Group 3 similarly decreased activity to 5.22 U/mg protein (49.4% decrease). Bacteriocin supplementation markedly restored GST activity, with Group 4 reaching 7.69 U/mg protein (25.6% recovery relative to control) and Group 5 improving to 7.74 U/mg protein (25.1% recovery). These findings indicate that *L. pentosus* C82 bacteriocin treatment effectively enhances GST activity, contributing to the restoration of detoxification and antioxidant defense mechanisms in dysbiosis-induced models.

#### LPO estimation

LPO, measured as MDA levels, exhibited significant alterations across the experimental groups as shown in Figure 5E. Group 1 showed the lowest MDA level (38.36 nmol/mg protein), indicating minimal oxidative damage under normal conditions. Gut dysbiosis markedly increased oxidative stress, with *E. coli*-induced Group 2 reaching 90.56 nmol/mg protein (136.1% increase) and *S. marcescens*-induced Group 3 rising to 81.45 nmol/mg protein (112.4% increase) compared to the control. Remarkably, bacteriocin treatment effectively reduced lipid peroxidation, with Group 4 showing 53.22 nmol/mg protein (70.0% recovery) and Group 5 displaying 58.17 nmol/mg protein (62.6% recovery). These findings demonstrate that *L. pentosus* C82 bacteriocin supplementation significantly mitigates dysbiosis-induced lipid peroxidation, thereby protecting cellular membranes from oxidative damage.

#### NO estimation

NO levels showed significant alterations across the experimental groups as depicted in Figure 5F. Group 1 displayed baseline NO levels of 8.79 μmol/mg protein, representing normal physiological conditions. Exposure to pathogenic bacteria significantly increased NO production, with Group 2 -induced by *E. coli* showing 13.05 μmol/mg protein (a 48.5% increase) and Group 3 -induced by *S. marcescens* showing 13.25 μmol/mg protein (a 50.8% increase) compared to the control. Treatment with bacteriocin significantly reduced this elevation, with Group 4 showing a decrease to 10.86 μmol/mg protein (30.2% recovery) and Group 5 decreasing to 10.43 μmol/mg protein (29.5% recovery). These results indicate that *L. pentosus* C82 bacteriocin effectively suppresses pathogen-induced nitric oxide overproduction, thereby alleviating inflammatory oxidative stress in dysbiosis-induced models.

#### Gene expression study

The gene expression profiling revealed that bacteriocin treatment notably modulated both host immune and gut microbial gene markers in zebrafish larvae (Figure 5G). The dysbiosis-induced groups (Groups 2 and 3) exhibited significant reductions in the expression of IL-10 (0.51- and 0.49-fold), tight junction genes Claudin 5a (0.49- and 0.52-fold) and ZO-1 (0.13- and 0.16-fold) compared to the control (Group 1). In contrast, proinflammatory TNF-*α* levels were significantly elevated (1.64- and 1.73-fold). Treatment with C82 bacteriocin (Groups 4 and 5) effectively upregulated IL-10 (0.82- and 0.76-fold) and tight junction genes (Claudin 5a, 1.03- and 1.04-fold; *ZO-1*, 0.96-fold) while downregulating TNF-α expression (1.29- and 1.33-fold). These results suggest that C82 bacteriocin exerts a gut-protective and anti-inflammatory effect by reinforcing epithelial integrity and balancing cytokines.

Similarly, oxidative stress-related genes demonstrated clear modulation upon bacteriocin treatment, as shown in Figure 5H. In dysbiosis groups, Nfe2l2a (encoding Nrf2) and *Hmox1a* were downregulated (0.53–0.54-fold and 0.54–0.58-fold), while bacterial biofilm-associated genes *csgD* and *bsmA* were upregulated (1.51–1.76-fold and 1.58-fold). Bacteriocin-treated groups (4 and 5) displayed significant recovery of antioxidant responses with increased Nfe2l2a (0.71- and 0.68-fold) and Hmox1a (0.48- and 0.76-fold) expression, along with a marked decline in *csgD* (1.25- and 1.33-fold) and *bsmA* (1.22- and 1.19-fold). These patterns indicate that C82 bacteriocin alleviates oxidative stress and suppresses biofilm-associated virulence.

Furthermore, gut microbiota-associated genes showed differential regulation across treatment groups, as shown in Figure 5I. In dysbiosis groups (2 and 3), *Lactobacillus* spp. and *Bifidobacteria* expression were significantly reduced (0.43–0.51-fold), whereas *Enterococcus* spp. levels increased (1.27–1.31-fold). Upon C82 bacteriocin treatment, the expression of *Lactobacillus* spp. (1.02–1.08-fold) and *Bifidobacteria* (0.91–0.95-fold) was restored, while *Enterococcus* spp. normalized (1.03–1.06-fold). These findings imply that bacteriocin from *L. pentosus* C82 promotes the recovery of beneficial commensal bacteria and stabilizes the gut microbial balance following dysbiosis.

## Discussion

In the present study, *Lactiplantibacillus pentosus* C82, isolated from fermented cabbage, was investigated for its antimicrobial, antioxidative, and gut-protective effects, with validation in a zebrafish model. The isolation rate of *Lactiplantibacillus* strains in this study (2.08 × 10^8^ CFU/mL) and the recovery of 90 distinct LAB isolates after 35 days of fermentation suggest cabbage as a good source of LAB. LAB counts from fermented vegetables typically range between 10^7^–10^8^ CFU/mL, with white cabbage and carrot yielding 2 × 10^7^–6 × 10^7^ CFU/mL and black carrot reaching up to 1.94 × 10^8^ CFU/mL at peak fermentation, which is compatible with the present findings (Lamba et al., 2019; Herman et al., 2024). Similarly, garlic-based fermentations occasionally exceed 10^8^ CFU/mL, suggesting our results are among the significant LAB densities observed in vegetable fermentations (Herman et al., 2024). Studies on cabbage and kimchi often report LAB densities of 10^7^–10^8^ CFU/mL with anywhere from 4 to over 100 isolates, depending on fermentation stage and culture methods (Begum et al., 2024). Collectively, these findings highlight that cabbage serves as a rich ecological niche for LAB proliferation and diversification. The isolation efficiency in this study reflects both the suitability of the substrate and the optimized fermentation conditions employed.

Among the six LAB isolates tested, strain C82 emerged as the most potent. It exhibited the largest inhibition zones of 1.5 cm and 13 cm against *E. coli* and *S. marcescens,* respectively, suggesting the production of antibacterial metabolites such as bacteriocin, short-chain fatty acid, and EPS. Previous studies have reported inhibition ranges of different *L. pentosus* strains, with pentocin MQ1 and TV35b, ranging from 8 to 22 mm (Wayah and Philip, 2018). Furthermore, the characterization of isolate C82 highlights unique phenotypic, functional, and molecular traits that collectively support its highest bacteriocin production and antibacterial activity. This strong activity can be attributed to the synergistic action of bacteriocin and EPS, as confirmed by its shiny, mucoid, and slightly ropy colony morphology. Previous reports have shown that EPS can enhance bacteriocin delivery and activity by disrupting pathogen biofilms, facilitating closer interactions with target cell membranes, and weakening bacterial defense systems (Soltani et al., 2022; Garza-Cervantes et al., 2023). The unusual co-occurrence of high bacteriocin yield and significant EPS production in C82 represents an antimicrobial synergy (Wang et al., 2020).

To determine if the antimicrobial activity of *Lactiplantibacillus pentosus* C82 was affected by lactic acid or other metabolic by-products, we conducted pH-neutralization and catalase-treatment assays. These assays are commonly used to distinguish bacteriocin-related inhibition from effects caused by acid or peroxide (Schillinger and Lcke, 1989; Todorov, 2009). Bacteriocins were the primary source of the zone of clearance. Neutralizing the CFS to pH 6.5 reduced the antimicrobial activity by removing the contribution of organic acids. In 2014, Yang et al. (2014) reported that organic acids can enhance LAB antimicrobial activity, but are not the sole factor. Importantly, some antimicrobial activity remained after neutralization, showing the presence of pH-independent antimicrobial compounds, typical of LAB-derived bacteriocins (Cleveland et al., 2001). Catalase treatment did not affect inhibitory activity, confirming that hydrogen peroxide was not involved. This supports previous studies indicating that catalase-resistant antimicrobial activity is due to bacteriocins rather than oxidative metabolites (Todorov and Dicks, 2005).

The biochemical characterization of C82 suggests stress tolerance at pH 2 and 0.5% bile salts, reinforcing its robustness as a probiotic, aligning with or surpassing probiotic benchmarks (Alizadeh Behbahani et al., 2025). Additionally, the catalase-negative and heterofermentative profile aligns with the classical characteristics of *Lactiplantibacillus,* while its ability to ferment multiple sugars (glucose, lactose, sucrose, and galactose) and produce lactic acid underscores its metabolic versatility and ecological fitness in the gut environment (Echegaray et al., 2023). The heterofermentative nature is particularly advantageous, as it generates lactic acid and other inhibitory metabolites such as acetic acid, ethanol, and CO₂, contributing to broad-spectrum antimicrobial activity and modulation of gut microbiota (Sinha et al., 2025). Moreover, molecular identification using 16S rRNA gene sequencing further confirmed the taxonomic placement, showing 99.76% sequence similarity with *Lactiplantibacillus pentosus*. It also demonstrated close homology with *L. plantarum* (99.67%) and *L. paraplantarum* (99.73%), echoing findings in recent genome mining of *L. pentosus* strains where bacteriocins were common despite genotypic closeness to *L. plantarum* (Soltani et al., 2022; Du et al., 2024). Taken together, the morphological, biochemical, and molecular analyses position C82 as a robust probiotic candidate. These traits not only distinguish C82 from conventional *L. pentosus* isolates but also highlight its application potential in therapeutic interventions targeting gut dysbiosis and biofilm-associated pathogens.

The optimization of bacteriocin production by *L. pentosus* C82 revealed a strong dependence on physiological and nutritional parameters, consistent with previous reports on LAB-derived antimicrobials. The incubation period was a critical determinant, with maximal bacteriocin activity observed at 72 h under shaking conditions, which coincided with the highest optical density and antibacterial activity. This pattern reflects the well-documented association between late exponential/early stationary phase metabolism and bacteriocin synthesis, as secondary metabolite production is typically triggered when primary growth stabilizes (Todorov and Dicks, 2006). In contrast, static conditions yielded significantly lower bacteriocin titers, emphasizing the importance of oxygen transfer and agitation for optimal metabolite secretion. Similar observations were reported for *Lactobacillus plantarum* ST26MS and *L. pentosus* strains, where aeration enhanced both growth and bacteriocin activity (Todorov and Dicks, 2005).

The impact of the carbon source on bacteriocin production was studied, as the carbon source is vital for LAB because it provides the fundamental building blocks (carbon molecules) for cell growth and energy, enabling the bacteria to synthesize essential cellular components. The choice of carbon source directly influences the rate of lactic acid production, the overall growth of the bacteria, and the production of other metabolites (Abedi and Hashemi, 2020). Among the tested carbohydrates, fructose was more effective than other sources in bacteriocin production. Fructose metabolizes via the phosphoenolpyruvate-dependent phosphotransferase system (PTS), allowing rapid uptake and glycolytic channeling, resulting in enhanced ATP flux and precursor availability for bacteriocin biosynthesis (Zhu et al., 2021). Additionally, prebiotic carbohydrates such as FOS and inulin exerted an even greater stimulatory effect. In *L. pentosus* C82, supplementation with 4% FOS and 5–6% inulin enhanced bacteriocin production. As inulin is hydrolysed by *β*-fructosidase into fructose units, it ensures a sustained carbon supply, supporting prolonged growth and secondary metabolite synthesis (Falony et al., 2009). Furthermore, carbohydrate-specific PTS transporters and operons such as fos RABCDXE regulate FOS/inulin utilization and have been implicated in activating bacteriocin gene clusters under favorable nutrient conditions (da Silva Sabo et al., 2015; Zhu et al., 2021). Previous studies confirmed that inulin supplementation not only enhances biomass accumulation but also modulates stress-response pathways, favoring antimicrobial metabolite yields (Falony et al., 2009). Another important source is nitrogen, as it is key for LAB production because it is an essential nutrient for cellular growth and metabolic activity. The control medium supported the highest yield, while supplementation with organic nitrogen resulted in only moderate levels. Inorganic nitrogen sources sharply suppressed bacteriocin biosynthesis. This aligns with reports that excess inorganic nitrogen exerts catabolite repression and pH shifts, which are unfavorable for bacteriocin expression (Prema et al., 2024). Organic nitrogen, in contrast, provides amino acid precursors but may divert metabolic energy toward primary growth rather than secondary metabolite synthesis (Todorov, 2009). Environmental stressors such as bile salts and pH also shaped bacteriocin yields in *L. pentosus* C82. Bile salts, at higher concentrations (≥0.2%) progressively suppressed bacteriocin activity, highlighting the sensitivity of LAB to bile-mediated membrane disruption (Begley et al., 2006). Similarly, C82 achieved optimal production at pH 4–6, which corresponds to the natural growth conditions of many LAB. Extreme acidic (pH 2–3) or alkaline (pH 8–10) environments sharply reduced bacteriocin yields, likely due to enzyme denaturation and impaired cell viability (Todorov and Dicks, 2005). Taken together, these results highlight that bacteriocin production in *L. pentosus* C82 is tightly regulated by nutrient availability, incubation dynamics, and environmental factors. Thus, the bacteriocin was produced using the optimized fermentation conditions.

Finally, the stability profile of the bacteriocin produced by *L. pentosus* C82 provides key insights into its potential for treating gut-related health issues. The results showed that *L. pentosus* C82 bacteriocin activity decreased at 60–80 °C and was fully inactivated at 100–120 °C. UV irradiation did not affect *L. pentosus* C82 bacteriocin activity significantly. Previous studies indicated that bacteriocin PB2 lost activity after 6 h of UV treatment, suggesting that *L. pentosus* C82 bacteriocin has features that resist photo-degradation (Otunba et al., 2022). The pH-dependent activity of *L. pentosus* C82 bacteriocin highlights its stability at pH 2–3, indicating that it could remain functional during stomach transit (Liang et al., 2025). The impact of heavy metals on bacteriocin activity showed that Zn^2+^ ions significantly enhanced the antimicrobial potency through various mechanisms, including membrane destabilization, inhibition of peptidoglycan biosynthesis, ROS generation, and structural stabilization of bacteriocin proteins (Abanoz and Kunduhoglu, 2018; Otunba et al., 2022). Regarding chemical stability, *L. pentosus* C82 bacteriocin was highly sensitive to strong denaturants, which disrupt protein secondary structure, while mild surfactants and alcohols partially preserved activity (Abanoz and Kunduhoglu, 2018). This resilience under moderate stress conditions suggests that *L. pentosus* C82 bacteriocin may be suitable for therapeutic formulations. Overall, the strong bacteriocin activity, along with exopolysaccharide production, antioxidant potential, and gastrointestinal resilience, indicate that *L. pentosus* C82 is a superior candidate among isolated strains and a unique probiotic with enhanced biotechnological and therapeutic applications especially against multidrug-resistant Gram-negative pathogens.

The partial purification of the bacteriocin from *L. pentosus* C82 using 70% ammonium sulfate precipitation followed by dialysis resulted in a significant improvement in purity. This was indicated by a 13.29-fold increase in specific activity and a 54.68% overall yield. This purification efficiency is consistent with previous observations where ammonium sulfate selectively concentrates cationic bacteriocins through salting-out mechanisms. In a study on *Lactococcus lactis* 63, a 16-fold purification with 82% yield was reported (Goyal et al., 2018). Although the yield of C82 was relatively lower, its higher specific activity (700 AU/mg) suggests effective enrichment of the active peptide. The presence of a single 16 kDa band in SDS-PAGE further confirms the successful removal of non-bacteriocin proteins. This is in contrast to earlier studies where low-molecular-weight bands (3.5–10 kDa) were predominant, as seen in pediocin-like and lactococcin-like bacteriocins. The higher molecular mass of the C82 bacteriocin suggests that it may belong to a different class of antimicrobial peptide with potentially broader functional domains or post-translational modifications. When compared to *L. pentosus* 124–2, which only showed a 3.15-fold purification and 0.41% yield despite two purification steps (Yuliana et al., 2023), the C82 bacteriocin demonstrated significantly better recovery and enrichment. This indicates that ammonium sulfate precipitation followed by dialysis is particularly effective for the C82 peptide, likely due to its physicochemical properties such as solubility, hydrophobic exposure, or net charge. The superior purification performance of C82 may also reflect the inherent robustness and stability of the molecule, similar to bacteriocins that have been shown to withstand heat and maintain activity under various pH conditions in comparative *Pediococcus* studies (Dey et al., 2019). Overall, these findings suggest that the *L. pentosus* C82 bacteriocin represents a distinct, higher-molecular-weight antimicrobial peptide with efficient recoverability, strong purification response, and promising functional stability. This makes it a valuable candidate for downstream characterization and biotechnological applications. The in-gel activation assay confirmed that the 16 kDa band observed on SDS-PAGE corresponds to the active bacteriocin, as the excised gel fragment produced clear inhibition zones against *E. coli* and *S. marcescens*. This directly links antimicrobial activity to the 16 kDa peptide and aligns with previous reports where in-gel assays validated the functional identity of purified bacteriocins. Similar to findings in *Bacillus subtilis* GAS101 (Sharma et al., 2018), where a 6.5 kDa band matched the active antimicrobial peptide, the C82 results verify the proteinaceous and electrophoretically stable nature of the bacteriocin.

The LC–MS/MS characterization of the active fraction from *L. pentosus* C82 provides strong evidence that the antimicrobial compound produced by this strain is peptide in nature. The late-eluting peaks at RT 10.232 and 11.118 min reflect a hydrophobic molecule typical of many Class II bacteriocins, which commonly elute in the mid-to-late region of reverse-phase gradients due to their amphipathic and membrane-active structures (Cotter et al., 2005; Drider et al., 2006). The MS/MS spectra revealed dominant fragment ions at m/z 475 and 549, together with multiple high-intensity ions between m/z 132–775, forming characteristic b- and y-type peptide fragment ladders that confirm backbone cleavage and thereby establish the proteinaceous identity of the antimicrobial agent. This fragmentation pattern is consistent with LAB-derived bacteriocins such as *plantaricins* and *pentocins*, which demonstrate a similar MS-based signature (Todorov, 2009; Perez et al., 2014). Moreover, the co-localisation of this peptide-rich fraction with a 16 kDa SDS-PAGE band that retains antimicrobial activity in the in-gel assay further supports its designation as a bacteriocin or BLIS. Comparable bacteriocin-like inhibitory substances have been documented in *L. pentosus* strains and are known for potent activity against Gram-negative pathogens such as *E. coli* and *Serratia* spp. (Cleveland et al., 2001; Mokoena, 2017; Darbandi et al., 2022). Collectively, these findings demonstrate that C82 produces a hydrophobic, proteinaceous antimicrobial peptide with biochemical characteristics closely aligned with those of established LAB bacteriocins, underscoring its potential application as a therapeutic antimicrobial.

Further, in understanding the potential of bacteriocins on gut dysbiosis, we evaluate their antioxidant activity through *in vitro* assays and a zebrafish dysbiosis model. The bacteriocin showed strong antioxidant potential in vitro, with DPPH radical-scavenging activity and ABTS inhibition. The results indicated a clear increase in scavenging activity with higher concentrations. These findings are consistent with previous studies that reported potent antioxidant properties of bacteriocins from other LAB and bacterial strains, such as those from *Paenibacillus polymyxa* ALC101, *Enterococcus faecium* GRD AA ALC102 (Krishna et al., 2022). Thus, the high radical-scavenging capacity of *L. pentosus* C82 bacteriocin highlights its potential as both an antimicrobial and antioxidant agent, suggesting therapeutic applications in oxidative stress-related conditions like gut dysbiosis and neurodegeneration, where ROS imbalance plays a crucial role in disease progression.

Additionally, we assessed the toxicity profile of the bacteriocin in zebrafish embryos, as they are efficient, transparent, and sensitive models for observation (Cebrián et al., 2019). The *in vivo* developmental toxicity assays showed that *L. pentosus* C82 bacteriocin was well tolerated up to 20 μg/mL, with embryos displaying normal development over 24–72 h. However, concentrations ≥25 μg/mL led to dose-dependent developmental abnormalities, including delayed hatching, pericardial edema, and reduced survival, establishing a safety threshold at ≤20 μg/mL. Previous studies have also reported varying safety profiles for LAB-derived bacteriocins, like nisin and pediocin, indicating structural and strain-specific differences in safety (Paiva et al., 2012). The mild toxicity observed at higher concentrations of *L. pentosus* C82 is in line with the pore-forming mechanism of bacteriocins, which disrupt ion balance and induce developmental stress. This dose-dependent effect is similar to findings for nisin and bovicin HC5. Importantly, the toxic concentrations of *L. pentosus* C82 are significantly lower than those reported for other bacteriocins like subticin 112 (>20 g/kg in animal models), suggesting that *L. pentosus* C82 has a narrower safety margin (Parada Fabián et al., 2025).

In assessing the antioxidant activity of bacteriocins on gut dysbiosis, the zebrafish larvae model was employed. The administration of *L. pentosus* C82 bacteriocin effectively restored antioxidant enzyme activities and mitigated oxidative stress markers in zebrafish models of gut dysbiosis, demonstrating its potent redox-modulatory potential. In the present study, SOD and CAT activity, which were markedly reduced by dysbiosis-inducing pathogens (*E. coli* and *S. marcescens*), showed near-complete recovery following bacteriocin supplementation. Specifically, Group 4 and Group 5 exhibited levels approaching those of the control group. This aligns with earlier reports that bacteriocin-producing probiotics, such as *Lactobacillus salivarius* S01, enhance SOD activity and protect against oxidative injury in zebrafish, indicating that bacteriocin-mediated microbial restoration may normalize ROS metabolism, while the recovery of CAT activity indicates effective detoxification of hydrogen peroxide, consistent with probiotic interventions shown to elevate catalase activity under oxidative stress conditions (Jiang et al., 2024). Moreover, GSH levels, a major indicator of cellular redox balance, exhibited substantial restoration with bacteriocin-treated groups, compared to the dysbiosis groups. Additionally, the increase in GSH aligned with other observations from bacteriocin and probiotic supplementation studies, suggesting elevated intracellular antioxidant capacity and reduced oxidative injury.

During GST activity, which plays a critical role in detoxification and conjugation reactions, was also enhanced by C82 bacteriocin. Dysbiosis induction was nearly reduced by 50%, which was further ameliorated by bacteriocin treatment, leading to significant recovery. The restored GST activity underscores the bacteriocin’s capacity to promote enzymatic defense against electrophilic and oxidative stressors (Lanzarin et al., 2023). In contrast, markers of oxidative damage, namely lipid peroxidation (LPO) and nitric oxide (NO) production, were markedly reduced following C82 bacteriocin treatment. This, provides direct evidence for bacteriocins reducing NO production in zebrafish gut dysbiosis, demonstrating the bacteriocin’s protective effect against ROS-induced membrane damage. Similarly, earlier studies reported that probiotic *Lactobacillus plantarum* SHY21-2, protected zebrafish against *Aeromonas hydrophila* infection by reducing oxidative damage and LPO (Jiang et al., 2024). These findings are in agreement with previous studies reporting that bacteriocin-producing LAB strains can downregulate inflammatory mediators and oxidative byproducts in zebrafish and other animal models.

Further evidence, through RT-qPCR gene expression, analysis, demonstrates the therapeutic activity of the bacteriocin. For example, treatment with bacteriocin resulted in the upregulation of IL-10 and downregulation of TNF*α*, indicating the promotion of anti-inflammatory pathways that lead to the suppression of an excessive immune response (Carlini et al., 2023). The upregulation of claudin 5α, and ZO-1 suggests the restoration of the barrier, preventing the translocation of pathogens (Zeng et al., 2025). Conversely, the downregulation of Nfe2l2a, Hmox1a, csgD, and bmsA emphasis the reduction in oxidative stress, and suppression of bacterial biofilm formation, respectively (Anjana and Tiwari, 2022). The decreased expression of all *Enterococcus* spp. indicates the antimicrobial activity of the bacteriocin (Zeng et al., 2025). Overall, these gene expression patterns demonstrate that *L. pentosus* C82 bacteriocin improves pathogen-induced gut dysbiosis by modulating inflammatory, oxidative, and microbial pathways, strengthening intestinal barrier integrity, enhancing antioxidant defense, and restoring a healthy microbiota composition.

This investigation primarily focused on short-term evaluation using zebrafish larvae. While effective for preliminary screening, zebrafish larvae may not entirely represent the complexity of mammalian gut systems. Therefore, the findings require validation in higher animal models to confirm translational relevance. Additionally, long-term studies assessing microbial diversity, host–microbe interactions, and safety profiles are warranted. Further biochemical characterization of the bacteriocin could also provide deeper insights into its mechanism of action and therapeutic applicability.

In the present study, MRS medium was used as the basal medium for optimizing bacteriocin production. However, because cabbage is naturally rich in cellulose and fermentable sugars, it represents a promising renewable carbon source that may reduce production costs while supporting sustainable bacteriocin biosynthesis. Future work will evaluate whether cabbage extract or cellulose hydrolysate can partially or completely replace MRS medium to enhance the growth and bacteriocin yield of *L. pentosus* C82. Beyond substrate optimization, Future studies will incorporate advanced microbiome sequencing approaches, including 16S rRNA sequencing and shotgun metagenomics, to obtain a comprehensive community-wide profile of gut microbial composition, diversity, and functional pathways, thereby enabling precise confirmation of gut microbial restoration beyond culture-based analyses. Although the present study demonstrates modulation of the GBA through cytokine regulation (IL-10, TNF-*α*), barrier restoration (ZO-1, Claudin-5a), oxidative-stress signaling (Nfe2l2a, Hmox1a), and behavioral improvement, future investigations will include neurochemical assessments such as dopaminergic markers (TH, drd1/drd2, DAT), serotonergic genes (tph2, slc6a4), neurotrophic factors (BDNF), α-synuclein expression, and brain-region-specific inflammatory and oxidative-stress markers to establish mechanistic GBA involvement. As a preliminary model, the present work utilizes zebrafish larvae; however, to enhance translational relevance, future studies will extend to cell culture systems (neuronal, microglial, and intestinal epithelial cells) to delineate mechanistic pathways, as well as mouse models of PD to validate behavioral, neurochemical, and microbiome outcomes in a mammalian system. Additionally, pharmacokinetic profiling of bacteriocin in mammalian hosts will be conducted to support preclinical development and potential therapeutic translation.

## Conclusion

In conclusion, *Lactiplantibacillus pentosus* C82, isolated from fermented cabbage, exhibits potent antimicrobial, antioxidative, and gut-protective properties, reinforcing its potential as a strong probiotic candidate. The strain produced high levels of bacteriocin under optimized conditions and demonstrated stability under acidic pH, bile salt exposure, and moderate heat stress. LC–MS/MS analysis further confirmed the peptide-based nature of the active antimicrobial molecule, supporting its classification as a bacteriocin/BLIS. Toxicity assessment in zebrafish embryos established its safety up to 20 μg/mL, while *in vivo* studies showed that C82 bacteriocin effectively restored antioxidant enzyme activities (SOD, CAT, GSH, GST) and reduced oxidative stress markers (LPO, NO) in gut dysbiosis models. Gene expression profiling additionally demonstrated its anti-inflammatory and barrier-protective roles through IL-10 upregulation and TNF-α suppression. Collectively, the safety profile of C82 and its ability to modulate gut-brain axis-related pathways highlight its promise as a next-generation probiotic and natural antimicrobial for managing dysbiosis, enhancing gut homeostasis, and supporting overall host health.

## Data Availability

All data generated or analyzed during this study are included in this article. Data for this article, including qPCR primers, are available at NCBI (NCBI: NM_001020785.2; NCBI: NM_212859.2; NCBI: NM_213274.1; NCBI: BI706952.1; NCBI: DQ298393.1; NCBI: AY365115.1; NCBI: HM007611.1; NCBI: PV012319.1; NCBI: AF537272.1; NCBI: NM_182889.1; NCBI: NM_001127516.1; NCBI: NM_131031.2) at https://www.ncbi.nlm.nih.gov/; IL10: https://www.ncbi.nlm.nih.gov/nuccore/NM_001020785.2; TNF-α: https://www.ncbi.nlm.nih.gov/nuccore/NM_212859.2; Claudin 5a: https://www.ncbi.nlm.nih.gov/nuccore/NM_213274.1; ZO-1: https://www.ncbi.nlm.nih.gov/nuccore/BI706952.1; all Bifidobacteria: https://www.ncbi.nlm.nih.gov/nuccore/DQ298393.1; all *Lactobacillus* spp.: https://www.ncbi.nlm.nih.gov/nuccore/AY365115.1; all *Enterococcus* spp.: https://www.ncbi.nlm.nih.gov/nuccore/HM007611.1; *csgD:* https://www.ncbi.nlm.nih.gov/nuccore/PV012319.1; *bsmA:* https://www.ncbi.nlm.nih.gov/nuccore/AF537272.1; Nfe2l2a: https://www.ncbi.nlm.nih.gov/nuccore/NM_182889.1; Hmox1a: https://www.ncbi.nlm.nih.gov/nuccore/NM_001127516.1; and Beta-actin: https://www.ncbi.nlm.nih.gov/nuccore/NM_131031.2.
